# Chimeric enzymes enhance treatment potential for globoid cell leukodystrophy through hematopoietic stem cell gene therapy

**DOI:** 10.1016/j.ymthe.2025.09.030

**Published:** 2025-09-22

**Authors:** Federica Cascino, Alessandra Ricca, Ilaria Picciotti, Erika Valeri, Giulia Unali, Veronica Saporito, Marta Freschi, Francesco Morena, Sabata Martino, Anna Kajaste-Rudnitski, Angela Gritti

**Affiliations:** 1 IRCCS San Raffaele Scientific Institute, San Raffaele Telethon Institute for Gene Therapy (SR-Tiget), 20132 Milan, Italy; 2 Research Institute of Molecular Pathology (IMP), 1030 Vienna, Austria; 3 National Emerging Infectious Diseases Laboratories (NEIDL), Boston University, Boston, MA 02118, USA; 4 Gene Therapy Program of Dana-Farber/Boston Children’s Cancer and Blood Disorder Center, Boston, MA 02215, USA; 5 Department of Chemistry, Biology, and Biotechnology, University of Perugia, 06122 Perugia, Italy; 6 Department of Biology and Biotechnology, University of Pavia, 27100 Pavia, Italy; 7 Vita-Salute San Raffaele University, 20132 Milan, Italy

**Keywords:** globoid cell leukodystrophy, chimeric enzymes, hematopoietic stem/progenitor cells, gene therapy, lentiviral vectors, cross-correction, Krabbe disease, metabolic diseases, stem cell transplant

## Abstract

Globoid cell leukodystrophy (GLD) is a fatal lysosomal storage disorder caused by a deficiency in the β-galactosylceramidase (GALC) enzyme, leading to severe demyelination and neurodegeneration, and often death before the age of 2 years. Hematopoietic stem/progenitor cell transplantation (HSPC-T) has limited efficacy due to inadequate GALC delivery to the central (CNS) and peripheral nervous systems (PNS) and associated risks. *In vivo* gene therapy (GT) using adeno-associated viral vectors shows promise, but safety concerns persist. This research presents a strategy using lentiviral (LV) vector-mediated *ex vivo* HSPC-GT with a chimeric GALC enzyme that incorporates peptides from α-l-iduronidase (IDUA) and apolipoprotein E II (APO) to enhance expression and blood-brain barrier penetration. The chimeric IDUAsp.GALC.APO enzyme exhibited superior production and secretion compared to native GALC and previous chimeric variants in LV-transduced HSPCs, resulting in improved cross-correction and normalization of GALC activity in GLD neural cells. Proof-of-concept studies demonstrated effective enzyme production, secretion, and cross-correction capability of macrophages from GLD patients. *In vivo* results showed stable gene marking, sustained enzyme production, and efficient delivery of the chimeric GALC in affected organs, including the CNS and PNS. These findings highlight the potential of HSPC-GT using chimeric GALC enzymes as an innovative therapeutic approach for treating GLD.

## Graphical abstract

**Figure:**
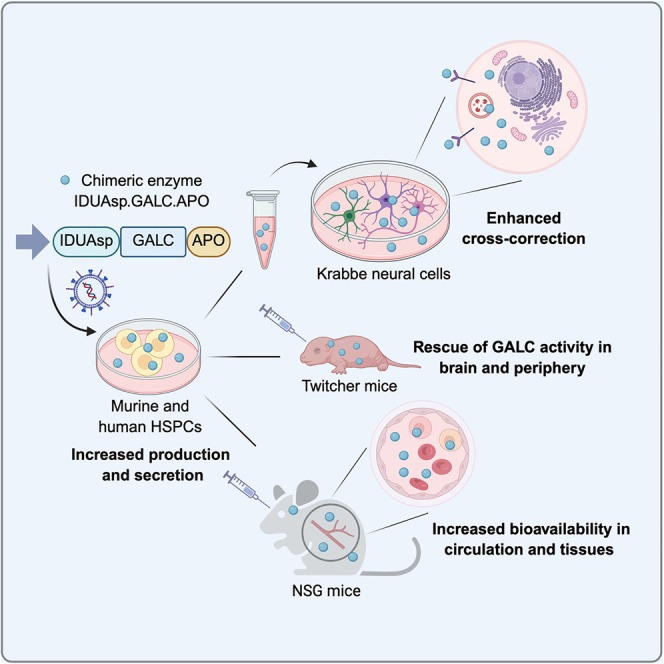

## Teaser

Gritti and colleagues developed a lentiviral vector expressing a chimeric GALC enzyme with enhanced production, secretion, and cross-correction. Their studies in murine models and patient-derived cells showed stable gene marking, increased bioavailability, and effective CNS and PNS delivery, demonstrating the therapeutic potential of chimeric GALC in HSPC-GT for GLD.

## Introduction

Globoid cell leukodystrophy (GLD, or Krabbe disease) is a severe lysosomal storage disorder (LSD) resulting from a deficiency of β-galactosylceramidase (GALC), an enzyme critical for degrading myelin galactolipids. This enzymatic deficiency leads to the pathological accumulation of substrates such as galactosylceramide (GalCer) and psychosine, triggering neuroinflammation, demyelination, and neurodegeneration in both the central (CNS) and peripheral nervous systems (PNS).^1^ The early infantile form of GLD is particularly aggressive, often resulting in death before the age of 2 years.^2^ The therapeutic options are limited for GLD, with no curative treatments currently available. Correcting protein deficiencies in early-onset LSDs is challenging due to the need for treatments that can quickly and effectively target multiple tissues. A significant obstacle is the blood-brain barrier (BBB), which restricts the delivery of therapeutic proteins to the brain.^3^^,^^4^^,^^5^

The only approved treatment for GLD is hematopoietic stem/progenitor cell transplantation (HSPC-T) using bone marrow (BM) or umbilical cord blood cells. When performed presymptomatically, it can extend lifespan and improve cognitive and motor functions.^6^^,^^7^^,^^8^^,^^9^ This approach aims to repopulate the patient’s hematopoietic system with donor cells that secrete functional lysosomal enzymes, which surrounding cells can take up through cross-correction.^10^ HSPC-T does not entirely prevent CNS and PNS degeneration due to inadequate GALC delivery and limited cross-correction of neurons and glial cells. Despite protocol improvements, HSPC-T still carries significant risks.^11^^,^^12^ Gene therapy (GT) using adeno-associated viral (AAV) or lentiviral (LV) vectors is being explored as an alternative to enhance GALC expression directly.

*In vivo* systemic and intracerebral GT using AAV vectors has shown promise in preclinical GLD models, leading to GALC overexpression, extended survival, and partial correction of pathological hallmarks.^13^^,^^14^^,^^15^ Recent clinical trials evaluate AAV-mediated GT combined with HSPC-T to treat GLD infants (NCT04693598 and NCT05739643). The administration of AAVs, primarily through systemic delivery, raises several safety concerns, including hepatic damage,^16^^,^^17^ dorsal root ganglion toxicity,^14^ and genotoxicity.^18^^,^^19^ These issues arise mainly when a substantial amount of AAV particles reaches peripheral organs due to the high doses required to achieve therapeutic levels in CNS tissues. This situation underscores the necessity for careful long-term monitoring of treated patients^20^ and emphasizes the ongoing efforts to enhance vector design and production.^21^^,^^22^

LV-mediated *ex vivo* HSPC-GT represents a promising alternative to *in vivo* approaches for the treatment of LSDs. This strategy enables sustained, high-level expression of therapeutic proteins from genetically modified autologous HSPCs. Upon reinfusion, these engineered cells successfully engraft within BM niches and differentiate into myeloid lineages, including macrophages and microglia-like cells, mediating long-lasting therapeutic effects in the PNS and CNS.^23^^,^^24^ The use of myeloablative conditioning to facilitate HSPC engraftment is a well-established clinical practice, characterized by predictable and manageable toxicity profiles.^25^ Furthermore, vector design and promoter optimization advancements have markedly decreased the genotoxicity risks traditionally associated with integrating vectors.^26^ Long-term clinical follow-up studies have further confirmed the safety and durability of this approach, demonstrating stable hematopoiesis without malignant transformation in treated patients.^27^ The successful application of LV-mediated HSPC-GT in disorders such as metachromatic leukodystrophy and mucopolysaccharidosis type I Hurler^25^^,^^28^ highlights its therapeutic potential for GLD, despite unique disease-specific challenges remaining.

Preclinical studies in Twitcher (TWI) mice, a model mimicking early-onset GLD,^29^^,^^30^ have shown limited benefits of HSPC-GT compared to allogeneic HSPC-T.^31^^,^^32^^,^^33^ Slow brain myeloid cell repopulation and insufficient GALC overexpression in HSPCs and their progeny^31^^,^^32^ limit the benefits of this approach in rapidly progressing forms that require urgent and widespread enzymatic rescue in the CNS. Additionally, the complex post-translational processing of GALC, essential for proper lysosomal targeting,^34^^,^^35^ complicates therapy. While other lysosomal enzymes like arylsulfatase A (ARSA), α-l-iduronidase (IDUA), or iduronate-2-sulfatase (IDS) can be safely overexpressed by more than 100 times normal levels in LV-engineered HSPCs,^25^^,^^28^^,^^36^^,^^37^^,^^38^ GALC typically reaches only 2–3 times normal levels,^39^^,^^40^ with regulation varying by species, tissue, and cell type.

Enhancing GALC production, secretion, and BBB penetration is critical to maximizing the therapeutic potential of HSPC-GT in GLD. Protein engineering strategies, including signal peptides (sp) for enhanced production and secretion and low-density lipoprotein receptor (LDLr)-binding domains for improved BBB transcytosis, have been shown to boost the therapeutic benefit of GT in murine models of neurodegenerative LSDs.^37^^,^^41^^,^^42^ *Ex vivo* HSPC-GT using autologous HSPCs engineered for myeloid-specific expression of chimeric IDS fused with apolipoprotein E II (APO)-derived binding domain is being evaluated in mucopolysaccharidosis type II (MPSII) patients (NCT05665166). Developing chimeric GALC enzymes with enhanced bioavailability is crucial for GLD treatment, although these approaches remain underexplored.^43^^,^^44^^,^^45^

Our research aims to pioneer HSPC-GT approaches for GLD using chimeric GALC enzymes. In a previous study, we developed LVs with modified murine *Galc* transgenes incorporating the sp from the highly secreted IDS enzyme and the APO domain (IDSsp.m*Galc*.APO). This chimeric enzyme demonstrated superior secretion by LV-transduced neural stem/progenitor cells (NPCs) and rescued GALC activity in GLD neurons and glia.^40^ The advantage of the IDSsp.mGALC.APO enzyme was less pronounced in HSPCs, highlighting the transgene- and cell-type-specific protein overexpression and secretion, necessitating further optimization.

To address these challenges, we developed LVs expressing murine and human chimeric GALC enzymes using the IDUAsp to enhance protein secretion. The chimeric enzyme (IDUAsp.GALC.APO) outperformed native GALC and the IDSsp.GALC.APO variant, achieving up to 7- and 15-fold the physiological enzymatic activity and improving secretion from LV-transduced murine (Lin^−^ [lineage negative]) and human (CD34^+^) HSPCs progeny, respectively. This led to superior cross-correction and normalization of GALC activity in patient-derived neural cells. Proof of concept (PoC) was further shown using GLD patient-derived macrophages, where LV.IDUAsp.h*GALC*.APO restored GALC activity and cross-corrected (XC) human GLD-induced pluripotent stem cell (iPSC)-derived neural progeny. *In vivo* studies demonstrated that human HSPCs transduced with LV.IDUAsp.h*GALC*.APO successfully engrafted in immunodeficient mice, leading to enhanced enzyme availability in circulation and delivery to CNS and peripheral tissues compared to the native counterpart. In a severe GLD mouse model, the rapid progression of the disease limited the opportunity to fully demonstrate the therapeutic benefits of the chimeric enzyme within the HSPC-GT approach. Nevertheless, complementary systems, including *in vitro* studies using murine and human cell models and *in vivo* experiments with immunodeficient mice, confirmed the effective production and delivery of the chimeric GALC enzyme across various tissues, including the CNS.

These findings underscore the feasibility and efficacy of LV-mediated HSPC-GT using chimeric GALC enzymes for GLD, highlighting the transformative potential of this approach and warranting further development.

## Results

### Development and *in vitro* validation of a highly secreted and BBB-targeting murine chimeric GALC enzyme

**Figure 1:**
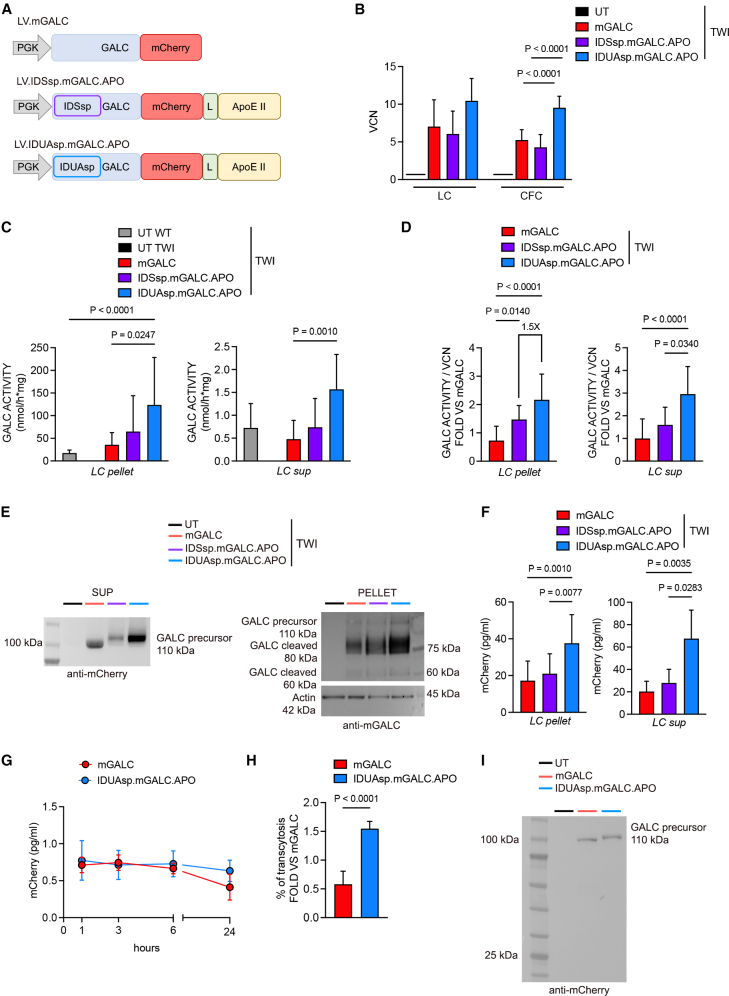
LV-transduced HSPCs overexpress and secrete the IDUAsp.mGALC.APOenzyme (A) Schematic of LVs encoding for m*Galc* and chimeric constructs (IDSsp.m*Galc*.APO and IDUAsp.m*Galc*.APO). *Galc* sequence fused with mCherry. APO, apolipoprotein E II receptor binding domain; IDSsp, sp sequence of iduronate-2-sulfatase enzyme; IDUAsp, sp sequence of α-l-iduronidase; L, linker. (B) VCN in TWI HSPCs transduced with the different LVs (100 MOI) and untreated (UT) TWI controls after 10 days (CFC) or 14 days (LC) in culture. Data are expressed as mean (SD) (*n* = 6–9 experiments, 2–3 technical replicates/experiment); one-way ANOVA, followed by Tukey’s multiple comparison test. UT values (black lines) are below the background threshold. (C) GALC activity in LC cultures (pellet and sup) from LV-transduced, UT WT, and TWI HSPCs. Data are the mean (SD), *n* = 6–9 experiments, 1–3 technical replicates/experiment; Kruskal-Wallis test, followed by Dunn’s multiple comparison test. (D) GALC activity normalized on the VCN in LC cultures from LV-transduced TWI HSPCs (pellet and sup). Data are expressed as fold vs. LV.m*Galc*-transduced TWI HSPCs. Data are expressed as the mean (SD), *n* = 6–9 experiments, 1–3 technical replicates/experiment; Kruskal-Wallis followed by Dunn’s multiple comparison test. In the pellet, the fold increase of IDUAsp.mGALC.APO to IDSsp.mGALC.APO is reported. (E) Representative WB showing (1) GALC precursor protein (anti-mCherry antibody) in the sup of UT TWI (negative control) and transduced-HSPC progeny (LC); (2) GALC precursor protein and cleaved form (anti-GALC antibody) in the pellet of UT TWI (negative control) and transduced-HSPC progeny (LC). (F) mCherry concentration (surrogate for GALC, measured by ELISA) in pellet and sup of transduced HSPC progeny (LC). Data are the mean (SD). Pellet: *n* = 4 experiments, with 3 biological replicates/experiment for pellets, 2 technical replicates. Sup: *n* = 2 experiments with 3 biological replicates/experiment, 2 technical replicates. One-way ANOVA, followed by Tukey’s multiple comparison test for pellet; Kruskal-Wallis, followed by Dunn’s multiple comparison test for sup. (G) mCherry concentration (surrogate for GALC, measured by ELISA) in the sup enriched with mGALC and IDUAsp.mGALC.APOenzyme. Data are the mean (SD) calculated as mCherry concentration (pg/mL) at 1, 3, 6, and 24 h of incubation divided by the mCherry concentration recorded immediately after plating (time 0). *n* = 2 experiments, 2 technical replicates/experiment. (H) Percentage of transcytosis of mCherry (surrogate for GALC, measured by ELISA) calculated as the ratio of mCherry after 24 h in the basolateral medium divided by mCherry at time 0, across a monolayer of bEND.3 endothelial cells in a transwell plate. Data represent the mean (SD), *n* = 5–6 experiments, 1–3 technical replicates/experiment. Unpaired Student’s *t* test. (I) Representative WB showing the GALC precursor protein (anti-mCherry antibody, 110 kDa) in the basolateral compartment of the transwell. Negative control: basolateral medium of a transwell incubated with the sup of UT TWI HSPC progeny (LC).

The sp are 5–30 amino acid sequences at the secretory proteins’ amino terminus region (N-region). The basicity of the N-polar region and the hydrophobicity of the hydrophobic (H)-core region positively impact protein production and secretion.^46^^,^^47^^,^^48^^,^^49^ In a previous study, we developed LVs with a modified *Galc* transgene incorporating the sp from the highly secreted IDS enzyme. The chimeric GALC enzyme demonstrated superior secretion by LV-transduced NPCs but only a moderate advantage in HSPCs.^40^ To enhance GALC biosynthesis and secretion from HSPCs for HSPC-GT applications, we screened sp sequences from various lysosomal enzymes using Peptide 2.0 software (Table S1). The IDUAsp showed the highest values of basicity of the N-region (43%) and hydrophobicity of the H-core (93.75%) compared to the sp of other lysosomal enzymes, including GALCsp (0% of basicity and 73,68% of hydrophobicity) and IDSsp (25% of basicity and 64.29% of hydrophobicity). We first generated vesicular stomatitis virus G protein (VSV-G)-pseudotyped third-generation LVs encoding a murine (m) GALC enzyme fused to the mCherry fluorescent tag to facilitate protein detection.^40^ Then, we engineered the construct by replacing the endogenous GALCsp with the IDUAsp and adding the APO-binding region to enhance BBB crossing^37^^,^^42^ and improve GALC uptake through LDLr and related proteins expressed in neural cells.^40^ The expression of the chimeric mGALC was driven by the ubiquitous human phosphoglycerate kinase (hPGK) promoter. We tested the chimeric GALC enzyme (IDUAsp.mGALC.APO) compared to the mCherry- tagged native GALC (mGALC) and the previously described IDSsp.mGALC.APO variant (Figure 1A). The modifications did not impact LV production, as shown by comparable titers and infectivities of different LV batches (Table S2).

We aimed to determine whether the IDUAsp provided an advantage over the native GALCsp and the previous IDSsp chimeric variant regarding GALC expression and secretion from HSPCs. We isolated lineage-negative (Lin^−^) HSPCs from the BM of 30- to 35-day-old TWI mice (fully symptomatic) and age-matched wild-type (WT) littermates. We transduced TWI HSPCs with the previously described LVs at 100 multiplicity of infection (MOI). We plated transduced cells and untreated (UT) TWI and WT controls for the colony-forming cell (CFC) assay and myeloid differentiation in liquid culture (LC) to evaluate GALC expression and potential toxicity related to the transduction procedure or transgene overexpression. We observed efficient HSPC transduction (Figure 1B) and vector copy number (VCN)-dependent supraphysiological GALC activity in the pellet and supernatant (sup) of LV-transduced HSPC progeny (LCs) (Figure S1A). The IDUAsp.mGALC.APO exhibited the highest GALC activity, reaching up to 7-fold (pellet) and 3-fold (sup) the physiological level assessed in UT WT HSPCs (Figure 1C). The LV transduction and the consequent GALC overexpression were safe, as demonstrated by a comparable number of colonies originating from UT and LV-transduced TWI HSPCs and UT WT counterparts (CFC assay; Figure S1B). Normalizing GALC activity to the VCN highlighted an increased intracellular enzymatic activity of the chimeric enzymes compared to mGALC and the advantage provided by IDUAsp.mGALC.APO compared to IDSsp.mGALC.APO (Figure 1D). The increased intracellular GALC activity correlated with the increased enzymatic activity in the sup (Figure S1C).

Intracellular GALC processing ensures proper lysosomal targeting and function. The GALC precursor protein (80 kDa) undergoes glycosylation and is trafficked to the lysosomes. In the acidic lysosomal environment, it is proteolytically cleaved into a 50-kDa N-terminal and a 30-kDa carboxyl-terminal subunit, which combine to form a functional enzyme complex.^34^^,^^50^ Qualitative confocal immunofluorescence (IF) and quantitative ImageStream analyses in LV-transduced HSPC progeny (LC) confirmed the lysosomal localization (LAMP1) of the chimeric IDUAsp.mGALC.APOenzyme (Figures S1D and S1E; proximity index = 88%), as previously described for mGALC and IDSsp.mGALC.APO counterparts.^40^

A fraction of the GALC precursor protein escapes the sorting pathway and is secreted in the extracellular space for uptake by surrounding cells in the cross-correction mechanism.^51^ Western blot (WB) analysis using an anti-mCherry antibody confirmed the presence of the GALC precursor protein (molecular weight ∼110 kDa, consisting of the 80 kDa GALC precursor fused to the 30 kDa mCherry tag) in the sup of LV-transduced HSPC progeny (Figure 1E). These findings suggest that similar to mGALC and IDSsp.mGALC.APO, the IDUAsp.mGALC.APO fusion protein is efficiently secreted, making it available for cross-correction of neighboring cells. Furthermore, WB analysis with an anti-GALC antibody detected both the precursor and cleaved forms of GALC in the LV-transduced HSPC progeny cell pellets, providing evidence for proper expression and intracellular processing of the enzyme (Figure 1E). To quantitatively evaluate enzyme production and secreted enzyme specifically available for cross-correction, we performed an ELISA detecting mCherry (a surrogate marker for GALC) in pellets and sup of LV-transduced HSPC progeny (LC), respectively. The analysis revealed a significant increase in the expression of the chimeric construct containing IDUAsp compared to the mGALC and IDSsp counterparts (Figure 1F).

The APO tag did not affect the half-life of the chimeric protein, as shown by the similar concentration of mCherry detected over 24 h in the sup of LV.m*Galc* and IDUAsp.m*Galc*.APO-transduced cells (Figure 1G).

The APO domain provided a significant advantage in the transcytosis of GALC in a simplified *in vitro* BBB model using bEND.3 endothelial cells in a transwell system (Figure 1H). This result confirms the potential for enhanced BBB penetration of APO-tagged lysosomal enzymes.^37^^,^^52^ Additionally, WB analysis with an anti-mCherry antibody revealed the presence of the GALC precursor protein fused to the mCherry tag (110 kDa) in the sup collected from the lower chamber of the transwell. In contrast, the mCherry tag alone (30 kDa) was undetectable (Figure 1I). The data confirmed the accuracy of the ELISA in detecting GALC precursor form, the variant potentially available for cross-correction.

The findings demonstrate that the chimeric IDUAsp.mGALC.APOenzyme exhibits superior intracellular expression and secretion compared to mGALC and IDSsp.mGALC.APO counterparts, with the APO domain enhancing transcytosis by endothelial cells without altering the enzyme’s half-life, highlighting the potential for increased enzyme bioavailability in HSPC-GT settings.

### Chimeric GALC enzymes secreted by HSPC progeny rescue GALC activity and reduce intracellular GalCer storage in TWI neural cells

**Figure 2:**
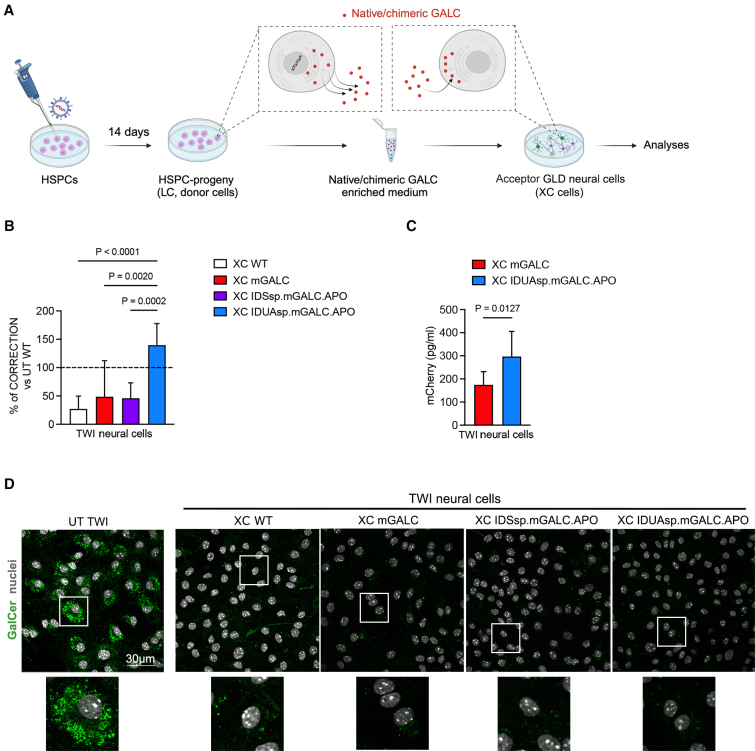
Chimeric GALC enzyme secreted by HSPC progeny rescues GALC activity, enhances uptake in acceptor cells, and reduces intracellular GalCer storage in TWI neural cells (A) Schematic representation of the cross-correction experiment. GALC-deficient neuronal/glial cell cultures (acceptor XC cells) are exposed for 24–72 h to the GALC-enriched sup collected from LV-transduced donor cells (HSPC progeny or CD14^+^-derived macrophages). At the end of the experiment, acceptor XC cells were collected for analyses. (B) GALC activity measured in acceptor TWI XC neural cells treated for 72 h with the sup collected from LV.m*Galc*-, LV.IDSsp.m*Galc*.APO-, and LV.IDUAsp.m*Galc*.APO-transduced HSPC progeny (donor cells). The enzymatic activity is the percentage of normal levels (measured in WT neural cells). Data are expressed as the mean (SD), *n* = 5–6 experiments, 1–2 technical replicates/experiment. One-way ANOVA followed by Tukey’s multiple comparison test. (C) Uptake of mCherry (a surrogate for GALC) by XC TWI neural cells exposed to the sup enriched with mGALC and IDUAsp.mGALC.APO. The ELISA measured mCherry concentration (pg/mL) in the cell lysate of acceptor cells. Data are expressed as the mean (SD), *n* = 3 experiments, 2 technical replicates/experiment, and analyzed by the Mann-Whitney test. (D) Representative confocal IF images and insets showing the reduction in GalCer storage (green) in TWI XC neural cells treated with the sup of donor cells compared to the UT TWI counterpart. Nuclei stained with Hoechst (gray, pseudocolor); *n* = 2–4 experiments, 2 coverslips/group/experiment; 63× magnification; scale bar: 30 μm.

To optimize *ex vivo* HSPC-GT, chimeric GALC enzyme released by GALC-overexpressing HSPC progeny should be internalized and transported to lysosomes of GALC-deficient neurons and glial cells. To compare the cross-correction capacity of the native and chimeric GALC enzymes, we cultured UT WT, LV.m*Galc*-, LV.IDSsp.m*Galc*.APO-, and LV.IDUAsp.m*Galc*.APO-transduced TWI HSPCs for 14 days (LC; donor cells, Figure 1B). The sup of donor cells collected every 24 h for the last 3 days of culture was used to treat UT TWI NPC-derived neural progeny (acceptor cells; 72 h of treatment) (Figure 2A). At the end of the experiment, we assessed GALC enzymatic activity, protein uptake, and GalCer storage in XC acceptor cells.

The sup collected from UT WT donors partially restored GALC activity in XC TWI neural cells, achieving ∼27% of the physiological levels (measured in WT neural cells). The sup from LV.m*Galc*- and LV.IDSsp.m*Galc*.APO-transduced donors significantly enhanced intracellular GALC activity in acceptor TWI cells, reaching ∼40%–50% of physiological levels. However, only the sup from LV.IDUAsp.m*Galc*.APO-transduced donors normalized GALC activity in acceptor TWI cells (Figure 2B). The increased GALC activity in the donor cell sup correlates with intracellular enzymatic activity observed in the XC cells (Figure S1F). Importantly, using equal GALC precursor input, we detected higher concentrations of mCherry in acceptor cells exposed to the sup enriched with IDUAsp.mGALC.APO protein (Figure 2C). This finding suggests a more efficient uptake by TWI neural cells, likely mediated by the expression of LDLr and related pathways.^40^

Confocal IF analysis demonstrated comparable clearance of GalCer storage in all XC cells (Figure 2D), indicating that ∼20% of the physiological intracellular enzymatic activity is sufficient to mediate substrate degradation under the *in vitro* culture conditions tested.^40^

These findings highlight the superiority of the chimeric IDUAsp.mGALC.APO in promoting GALC uptake and restoring enzymatic activity in XC TWI neural cells. Its potential to enhance the efficacy of HSPC-GT for GLD warrants further investigation in human-relevant in vitro models.

### Exploiting chimeric GALC enzymes for *ex vivo* HSPC-GT in TWI mice

We evaluated the safety and efficacy of HSPC-GT using chimeric GALC enzymes in TWI mice, a GLD model resembling the severity of the infantile GLD forms. Considering the early postnatal psychosine storage in TWI nervous tissues,^53^^,^^54^ we envisaged performing HSPC transplantation at postnatal days (PND) 2–3 following busulfan (BUS) conditioning. BUS was selected over total body irradiation (TBI) since it reduced tissue inflammation (Figure S2A), improved GALC activity restoration (Figure S2B), and enhanced survival (Figure S2C) with a lower engraftment rate (Figure S2D).

**Figure 3:**
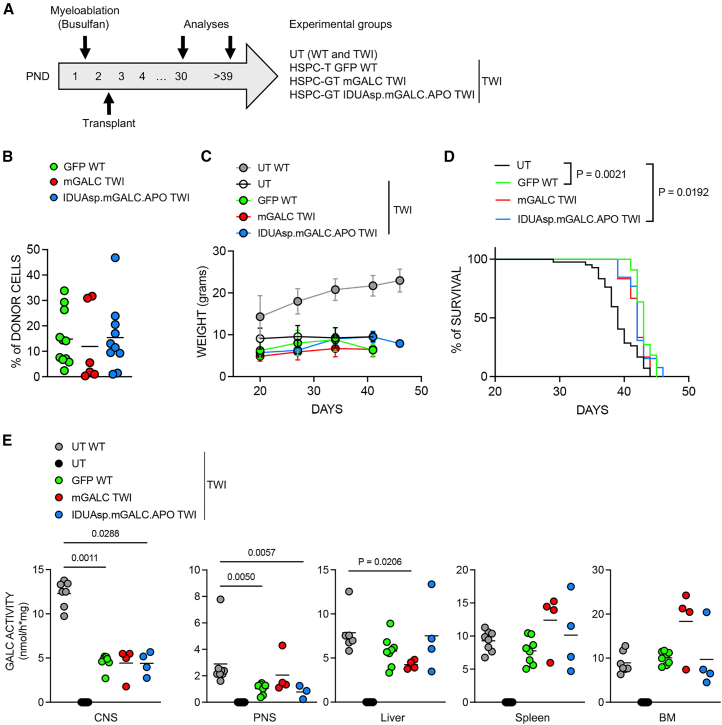
HSPC-GT in neonatal TWI mice using a chimeric enzyme (A) Experimental plan. Neonatal (PND1–2) TWI and WT mice are myeloablated by a single intraperitoneal injection of BUS (20 mg/kg). Transplantation of LV-transduced WT HSPCs expressing GFP (HSPC-T GFP WT) or LV-transduced TWI HSPCs expressing mGALC (HSPC-GT mGALC TWI) or IDUAsp.mGALC.APO (HSPC-GT IDUAsp.mGALC.APO TWI) is performed the following day. Treated TWI mice and age-matched UT WT and UT TWI controls are analyzed 1 month after transplant and at the end of the experiment (humane endpoint; >39 days, the average lifespan of UT TWI mice). (B) Percentage of donor-derived CD45^+^ cells (GFP^+^ WT HSPCs; mCherry^+^ TWI HSPCs) measured in the PB of treated TWI mice 1 month after transplant. Data are expressed as the mean. Each dot represents one mouse. (C) Body weight of treated TWI mice (GFP WT: *n* = 11, mGALC: *n* = 6, IDUAsp.mGALC.APO: *n* = 8), UT WT (*n* = 10), and UT TWI (*n* = 14), registered starting at 20 days of age. (D) Kaplan-Meier survival curves showing the survival percentage of treated and UT TWI mice. UT TWI: *n* = 40, GFP WT: *n* = 11, mGALC: *n* = 6, and IDUAsp.mGALC.APO: *n* = 8. Data analyzed using the log rank (Mantel-Cox) test. (E) Enzymatic GALC activity measured in CNS tissues (brain and spinal cord), PNS tissue (sciatic nerve), peripheral organs (liver and spleen), and BM of treated mice and UT controls (WT and TWI) at the end of the experiment. Data are expressed as the mean (SD). *n* = 2–3 experiments; each dot represents one mouse. Kruskal-Wallis test followed by Dunn’s multiple comparison test vs. UT WT.

We transplanted neonatal TWI mice with TWI Lin^−^ HSPCs transduced with LVs encoding m*Galc* constructs (Figure 3A). As control, transplantation using WT Lin^−^ HSPCs transduced with the LV.GFP (green fluorescent protein) was employed (VCN ∼6 in LC). The GALC activity of LC cultures from LV.GFP-transduced cells (∼24 nmol/h × mg) were comparable to UT LC counterparts (∼22 nmol/h × mg). The GALC activity of LV-transduced Lin^−^ TWI HSPC progeny is shown in Figure 1B. The average engraftment in the peripheral blood (PB) of BUS-conditioned transplanted mice was ∼15%, regardless of the treatment (Figure 3B), lower than reported for PND7–9 TWI mice receiving total bone marrow (tBM) from WT donors after TBI conditioning.^54^ We applied the BUS protocol for tBM transplantation (tBM-T) in PND2–3 TWI mice to exclude potential technical issues, achieving ∼50% engraftment (Figure S3A). We observed modest engraftment of HSPC-derived myeloid cells in the CNS of HSPC-transplanted mice (Figure S3B). This correlates with the reduced survival of HSPC-transplanted mice compared to tBM-T counterparts (Figure S3C), highlighting the difficulties in achieving effective HSPC engraftment in this model.

TWI mice with HSPC-GT treatment showed lower body weight than WT mice (Figure 3C). They experienced a modest increase in average lifespan (42 days) compared to TWI controls (39 days) (Figure 3D). The GALC activity was restored to normal or even supranormal levels in the BM, liver, and spleen, the first organs reached by transplanted HSPCs. We observed a substantial restoration of GALC activity in the PNS and CNS of transplanted mice, achieving up to 70% and 40% of WT levels, respectively (Figure 3E).

**Figure 4:**
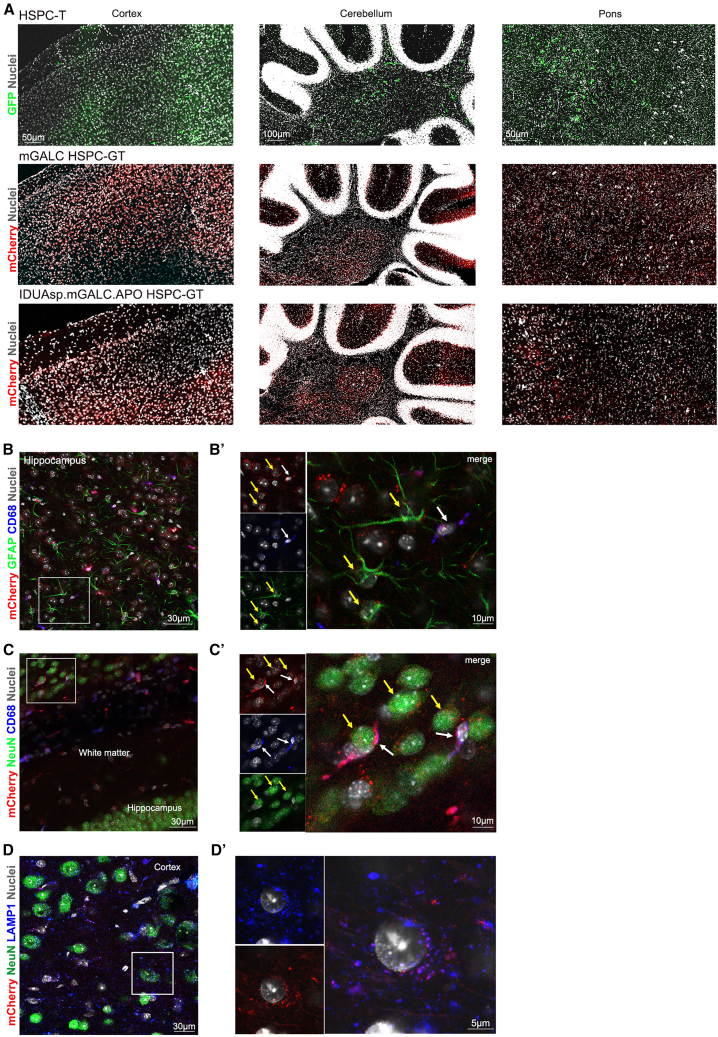
Engraftment of HSPC myeloid progeny in the brain of HSPC-GT-treated TWI mice and *in vivo* cross-correction (A) Representative fluorescence images of sagittal brain slices showing the distribution of engrafted mCherry^+^ cells (red) in the brains of HSPC-GT IDUAsp.mGALC.APO TWI analyzed at PND43. Nuclei stained with Hoechst (gray, pseudocolor); 20× magnification; scale bars: 50 and 100 μm. (B) Representative fluorescence images show the chimeric GALC enzyme’s expression (mCherry^+^ signal, red) in LV.IDUAsp.m*Galc*.APO HSPC-derived myeloid progeny (CD68^+^ cells, blue) and its uptake by GALC-deficient astrocytes (GFAP^+^ cells, green). Hippocampal region; 40× magnification; scale bar: 30 μm; *n* = 3 sagittal sections. (B′) Magnification of the image in (B). Scale bar: 10 μm. (C) Representative fluorescence images show the chimeric GALC enzyme’s expression (mCherry^+^ signal, red) in LV.IDUAsp.m*Galc*.APO HSPC-derived myeloid progeny (CD68^+^ cells, blue) and its uptake by GALC-deficient neurons (NeuN^+^ cells, green). Hippocampal region; 40× magnification; scale bar: 30 μm; *n* = 3 sagittal sections. (C′) Magnification of the image in (C). Scale bar: 10 μm. (D) Representative fluorescence image showing the correct lysosomal (LAMP1^+^ signal, blue) of chimeric IDUAsp.mGALC.APO enzyme (mCherry^+^ signal, red) in the GALC-deficient neurons (NeuN^+^ cells, green). Cortical region; 40× magnification; scale bar: 30 μm; *n* = 3 sagittal sections. (D′) Magnification of the image in (D); scale bar: 5 μm.

IF analysis showed that LV-transduced HSPC-derived progeny engrafted throughout the brain parenchyma of recipient TWI mice (Figure 4A; GFP^+^ and mCherry^+^ cells). The chimeric enzyme was correctly produced and secreted by LV-transduced CD68^+^ myeloid progeny and was efficiently recaptured by GFAP^+^ astrocytes (Figures 4B–B′) and NeuN^+^ neurons (Figures 4C–C′), where it localized in lysosomes (LAMP1^+^; Figures 4D–D′). These findings suggested that donor-derived myeloid progeny effectively XC GALC-deficient neuronal and glial cells. To establish PoC for *in vivo* cross-correction, we employed TWI mice that had undergone tBM-T, a model previously shown to display more consistent donor cell engraftment. This approach was chosen to overcome the limitation observed in HSPC-GT-treated mice, in which the low brain chimerism resulted in insufficient yields of engrafted CD45^+^ cells for reliable isolation and downstream analysis. In brain tissues from tBM-T TWI mice, we detected GALC enzymatic activity in CD45^−^ cells—presumed endogenous deficient brain cells—at approximately 30% of WT levels, indicative of effective cross-correction (Figure S3D). These findings suggest that HSPC-GT effectively delivers functional GALC enzymes to the affected areas despite modest donor chimerism in hematopoietic and brain tissues. This results in a substantial, even if partial, restoration of GALC activity in the CNS and PNS, key therapeutic targets in GLD, and complete enzymatic correction in peripheral organs.

### LVs expressing chimeric hGALC enzymes mediate effective and safe gene transfer in human CD34^+^ HSPCs and CD14^+^ monocytes

**Figure 5:**
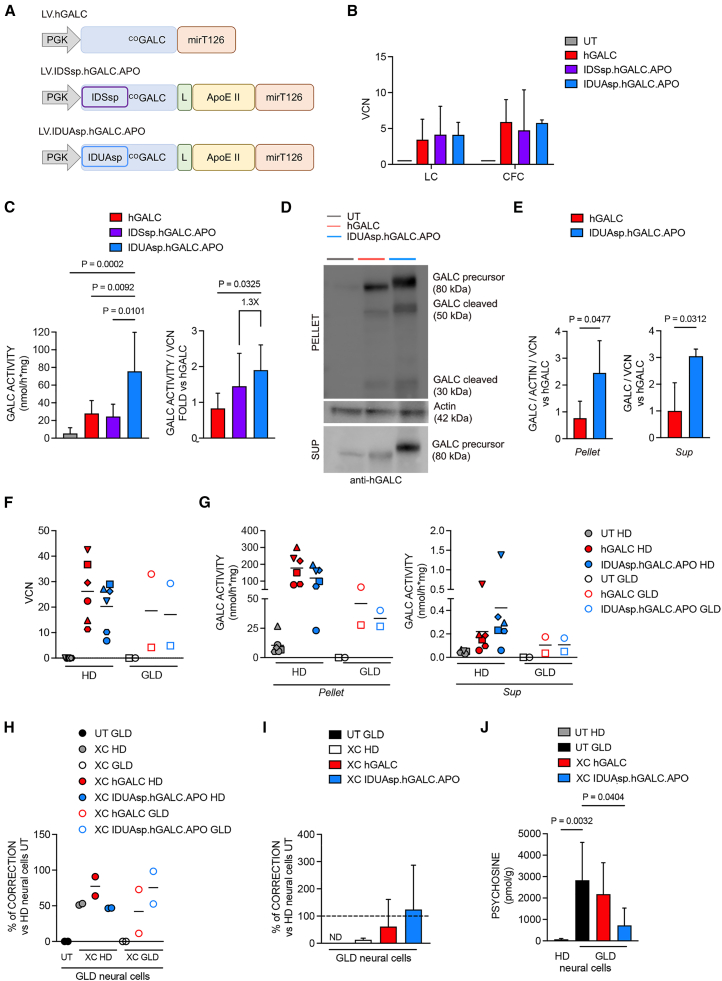
Robust and safe LV-mediated gene transfer in human CD34^+^ HSPC and CD14^+^ progeny and efficient cross-correction of human GLD neural cells (A) Schematic of LV encoding for h*GALC* and h*GALC* chimeric constructs (LV.IDSsp.h*GALC*.APO and LV.IDUAsp.h*GALC*.APO). The microRNA tag 126 (mirT126) was included in the original codon-optimized h*GALC* sequence.^44^ (B) VCN in CD34^+^ HSPCs transduced with LV.h*GALC*, LV.IDSsp.h*GALC*.APO, LV.IDUAsp.h*GALC*.APO, LV.GFP (100 MOI), and UT HD control after 14 days (CFC, LC) in culture. Data are expressed as the mean (SD), *n* = 5–6 independent experiments, 2 technical replicates/experiment. UT values (black lines) are below the background threshold. (C) GALC activity measured in pellets of UT and LV-transduced HSPC progeny (LC). Data are expressed as the mean (SD); *n* = 5–6 experiments, 2 technical replicates/experiments. One-way ANOVA followed by Tukey’s multiple comparison test; GALC activity (normalized on the VCN) measured in pellets of UT and LV-transduced HSPC progeny (LC). Data are expressed as the mean (SD), *n* = 5–6 experiments, 2 technical replicates/experiment. One-way ANOVA followed by Tukey’s multiple comparison test. The fold increase of IDUAsp.hGALC.APO to IDSsp.hGALC.APO is reported. (D and E) Representative WB (D) and quantification (E) showing GALC precursor protein (80 kDa) and processed forms (50 and 30 kDa) in pellet and sup of UT and LV-transduced CD34^+^ HSPC progeny (LC). Actin was used as a normalizer in pellets. Data in (E) are expressed as GALC/ACTIN/VCN in pellets and GALC/VCN in sup; mean (SD); *n* = 3 experiments, 1 technical replicate/experiment. Unpaired Student’s *t* test. (F) VCN measured 9 days post-transduction in HD and GLD CD14^+^-derived macrophages; *n* = 6 HD, *n* = 2 GLD. Data are expressed as the mean, *n* = 3 experiments, 1–2 technical replicates/experiment. Each shape represents 1 donor (legend in G). (G) GALC activity in UT and LV-transduced CD14^+^-derived macrophages (pellets and sup). *n* = 6 HD, *n* = 2 GLD. Data are expressed as the mean; *n* = 3 experiments, 1–2 technical replicates/experiment. Each shape represents 1 donor. (H) GALC activity in XC GLD hiPSC-derived neural acceptor cells treated for 24 h with the sup from UT and LV-transduced HD and GLD CD14^+^-derived macrophages (donor cells). The enzymatic activity is expressed as a percentage of the normal level (measured in HD hiPSC-derived neural cells). *n* = 2 experiments, 1–2 technical replicates. (I) GALC activity in XC GLD hiPSC-derived neural acceptor cells treated for 24 h with the sup from UT and LV-transduced HD CD34^+^ HSPC progeny (donor cells). The enzymatic activity is expressed as a percentage of the normal level (measured in HD hiPSC-derived neural cells). Mean (SD); *n* = 3–6 experiments, 1–2 technical replicates/experiment. (J) Psychosine content in XC GLD iPSC-derived neural acceptor cells exposed for 24 h to the sup of LV-transduced HD CD34^+^ HSPC progeny (donor cells). Data are expressed as mean (SD); *n* = 3 experiments, 1–2 replicates/experiment. One-way ANOVA followed by Tukey’s multiple comparison test.

We evaluated the efficacy and safety of chimeric human GALC (hGALC) enzymes in clinically relevant human hematopoietic cells. We used a VSV-G-pseudotyped third-generation LV backbone previously used in the HSPC-GT clinical trial for MPSI.^25^ We engineered a codon-optimized h*GALC* sequence^32^ (LV.h*GALC*) by replacing the GALCsp with the IDSsp and IDUAsp and included the APO sequence to generate LV.IDSsp.h*GALC*.APO and LV.IDUAsp.h*GALC*.APO (Figure 5A). These modifications are intended to enhance enzyme production, secretion, and targeting efficiency, similar to the murine version.

Pilot transductions in BM-derived CD34^+^ HSPCs from healthy donors (HDs) using LV.GFP and LV.IDSsp.h*GALC*.APO (50 and 100 MOI) with cyclosporine H (CsH) as a transduction enhancer^55^ determined the optimal culture conditions for maximizing VCN and GALC overexpression without cytotoxic effects. CsH improved transduction efficiency compared to the vehicle (DMSO) at 5 days post-transduction (Figure S4A). This resulted in increased VCN and GALC activity in HSPC-derived progeny (LC) at 14 days post-transduction (Figures S4B and S4C), with no observed toxicity related to LV transduction, CsH treatment, or GALC overexpression (Figure S4D).

Based on these results, we selected a dose of 100 MOI to transduce HD CD34^+^ HSPCs from mPB with LV.h*GALC*, LV.IDSsp.h*GALC*.APO, and LV.IDUAsp.h*GALC*.APO (Figure 5A) in the presence of CsH. The transduction was efficient, resulting in VCN-dependent GALC activity (Figures 5B and S5A) and an increase in mRNA expression of the exogenous h*GALC* transgene while the levels of the endogenous h*GALC* remained stable (Figure S5B). The transduction procedure and transgene expression did not adversely affect cell growth, colony formation (Figures S5C and S5D), or the composition of LC and CFC populations (Figures S5E–S5F) when comparing LV-transduced cells to UT CD34^+^ HSPC progeny. Furthermore, the LV transduction led to significantly elevated levels of GALC activity compared to physiological levels found in UT cells, with the IDUAsp.hGALC.APO variant exhibiting a remarkable 15-fold increase over normal levels (Figure 5C). Notably, IDUAsp.hGALC.APO outperformed hGALC and showed a 1.3-fold increase in GALC activity normalized for VCN compared to IDSsp.hGALC.APO (Figure 5C). Consequently, we selected LV.IDUAsp.h*GALC*.APO for further characterization.

WB analysis confirmed the correct production and secretion of the IDUAsp.hGALC.APO precursor protein (80 kDa) and the presence of the cleaved forms (50 and 30 kDa) (Figure 5D). The IDUAsp.hGALC.APO was produced and secreted more than the hGALC protein (Figure 5E). Confocal IF analysis confirmed GALC overexpression (Figure S5G) and the presence of the IDUAsp.hGALC.APO enzyme in lysosomes of HSPC-derived progeny (Figure S5H). We evaluated the effectiveness of LV.IDUAsp.h*GALC*.APO in restoring GALC enzymatic activity in patient-derived macrophages, a relevant model for assessing the therapeutic potential of the chimeric GALC enzyme in the context of HSPC-GT. We successfully transduced CD14^+^ monocytes isolated from the PB of two late-infantile GLD patients and six HDs using either LV.h*GALC* or LV.IDUAsp.h*GALC*.APO (Figure 5F). The transduced monocytes were differentiated into macrophages following an established protocol.^56^ After 9 days in culture, LV-transduced HD macrophages showed GALC overexpression at ∼11-fold the physiological levels. Notably, HD macrophages were transduced with LV.IDUAsp.h*GALC*.APO showed a 2-fold increase in extracellular GALC activity compared to those transduced with LV.h*GALC*. In macrophages derived from GLD patients, GALC activity was restored to physiological or supraphysiological levels following LV transduction (Figure 5G).

These *in vitro* findings demonstrated the safety and enhanced intra- and extracellular GALC activity of the chimeric IDUAsp.hGALC.APOenzyme by LV-transduced HSPC progeny, establishing it as a promising candidate for the HSPC-GT approach in treating GLD.

### Chimeric hGALC enzymes secreted by human CD14^+^ and CD34^+^ HSPC progeny cross-correct GLD-derived neural cells

We evaluated the cross-correction potential of native hGALC and chimeric IDUAsp.hGALC.APO, produced by engineered CD14^+^-derived macrophages and CD34^+^ HSPC progeny (donor cells, shown in Figures 5C and 5G) in GALC-deficient neuronal/glial mixed cultures from patient-iPSCs,^57^ exposed for 24 h to sup collected from LV-transduced donor cells.

The sup from LV-transduced GLD macrophages partially restored GALC activity in acceptor cells (XC), achieving up to normal values observed in HD human iPSC (hiPSC)-derived neural cells (Figure 5H). Notably, the sup from LV.h*GALC*-transduced CD34^+^ HSPC progeny restored GALC activity to 70% of HD values in GLD neural cells. GALC activity reached physiological levels in cells treated with IDUAsp.hGALC.APO-enriched sup (Figure 5I). A functional GALC enzyme is necessary for degrading psychosine, a toxic lipid accumulating in the brain tissues of GLD patients and GLD hiPSC-derived neural cells.^57^ The cross-correction mediated by IDUAsp.hGALC.APO was associated with a significant reduction in psychosine levels within 24 h of treatment in GLD XC neural cells (Figure 5J).

These findings demonstrate that both the hGALC and chimeric IDUAsp.hGALC.APO enzymes, secreted by transduced human HSPC progeny and macrophages, restore GALC activity in GLD human neural cells *in vitro*. The chimeric IDUAsp.hGALC.APO enzyme showed superior efficacy in reducing psychosine accumulation in this disease-relevant CNS model, underscoring its potential to enhance the therapeutic efficiency of HSPC-based GT for GLD.

### LV.IDUAsp.h*GALC*.APO-transduced CD34^+^ HSPCs engraft in immunodeficient NOD scid gamma mice and release the chimeric GALC enzyme

**Figure 6:**
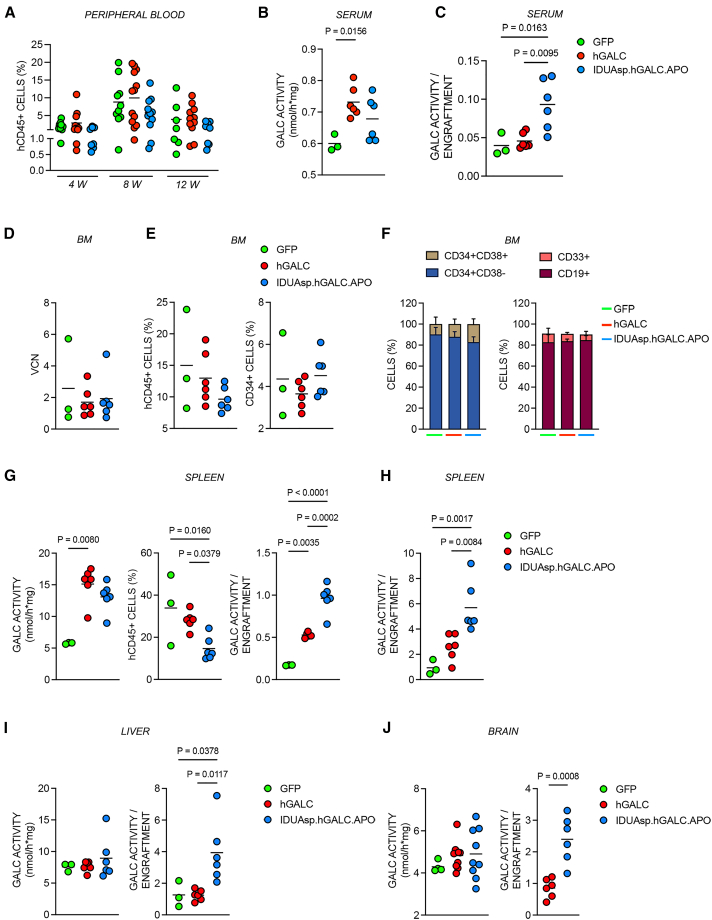
Persistent gene transfer and enhanced bioavailability upon xenotransplantation of LV-transduced CD34^+^ HSPCs in NSG mice (A) Engraftment levels (percentage of hCD45^+^ cells) in the PB of NSG mice at 4, 8, and 12 weeks post-transplantation. Data are expressed as the mean; *n* = 3 experiments; each dot represents one mouse (legend in C). (B) GALC activity in the sera of transplanted NSG mice at 8 weeks post-transplant. Data are expressed as the mean. One-way ANOVA, followed by Tukey’s multiple comparison test. *n* = 1 experiment, each dot represents one mouse (legend in C). (C) GALC activity in the sera normalized on the percentage of engraftment in PB at 8 weeks. Data are expressed as the mean. One-way ANOVA, followed by Tukey’s multiple comparison test. *n* = 1 experiment; each dot represents one mouse. Fold change: IDUAsp.hGALC.APO vs. hGALC, 2×; IDUAsp.hGALC.APO vs. GFP, 2.3×; GALC vs. GFP, 1.2×. (D) VCN measured in the BM of transplanted NSG mice at the time of sacrifice (16 weeks). Data are expressed as the mean. *n* = 2 experiment; each dot represents one mouse (legend in E). (E) Percentage of hCD45^+^ (left) and hCD34^+^ cells (right) in the BM of transplanted NSG mice. Data are expressed as the mean. *n* = 1 experiment; each dot represents one mouse. (F) Cell composition (expressed in percentage of total cells) in the BM of transplanted NSG mice at the time of sacrifice (16 weeks); GFP: *n* = 3 mice, hGALC: *n* = 6 mice, IDUAsp.hGALC.APO: *n* = 6 mice. Progenitors (CD34^+^CD38^+/−^, left), myeloid cells and B lymphocytes (CD33^+^ and CD19^+^, respectively, right) are represented in different colors. Data are expressed as the mean (SD), *n* = 1 experiment. (G) Analyses on the spleen of transplanted NSG mice at 16 weeks: GALC activity (left), data are expressed as the mean and analyzed by Kruskal-Wallis test followed by Dunn’s multiple comparison test; percentage of hCD45^+^ cells (center) and GALC activity normalized on the percentage of engrafted hCD45^+^ cells (right); data are expressed as the mean and analyzed by one-way ANOVA, followed by Tukey’s multiple comparison test. *n* = 1 experiment; each dot represents one mouse (legend in H). (H) GALC activity in the spleen normalized on the percentage of engraftment in the PB at 12 weeks. Data are expressed as the mean. One-way ANOVA followed by Tukey’s multiple comparison test. *n* = 1 experiment; each dot represents one mouse. (I) Analyses on the liver of transplanted NSG mice at 16 weeks: absolute GALC activity (left) and normalized on the percentage of engraftment in the PB at 12 weeks (right). Data are expressed as the mean and analyzed by one-way ANOVA, followed by Tukey’s multiple comparison test. *n* = 1 experiment; each dot represents one mouse. (J) Analyses on the brain of transplanted NSG mice at 16 weeks: absolute GALC activity (left) and normalized on the percentage of engraftment in the PB at 12 weeks (right). Data are expressed as the mean and analyzed using the unpaired Student’s *t* test. *n* = 2 experiments; each dot represents one mouse.

To assess the safety of *in vivo* production and secretion of chimeric hGALC enzymes in HSPC-GT, we transplanted mobilized PB (mPB)-derived HD CD34^+^ cells transduced with LV.IDUAsp.h*GALC*.APO, LV.h*GALC*, or LV.GFP (used as control; VCN ∼3) into irradiated female immunodeficient NOD scid gamma (NSG) mice. We monitored the presence of genetically corrected circulating human cells by performing flow cytometry analysis on PB samples collected monthly from the treated mice, starting 4 weeks after transplantation. The results showed a gradual increase in human hematopoietic cell engraftment (hCD45^+^), peaking at 8 weeks, with a 20% chimerism. Cell engraftment moderately decreased by 12 weeks, with no significant differences observed among the treatment groups at any time (Figure 6A).

We observed increased GALC enzymatic activity in the serum of mice transplanted with LV.IDUAsp.h*GALC*.APO- and LV.h*GALC*-transduced cells compared to those transplanted with LV.GFP-transduced cells (controls) at 8 weeks (Figure 6B). Normalizing the serum enzymatic activity to the PB engraftment highlighted superior secretion and enhanced enzyme bioavailability in the bloodstream of mice receiving LV.IDUAsp.h*GALC*.APO-transduced compared to those receiving LV.h*GALC*-transduced cells (Figure 6C).

At the time of sacrifice (16 weeks), the VCN in the BM of treated mice confirmed persistent engraftment and gene marking (Figure 6D). We detected comparable levels of hCD45^+^ and hCD34^+^ cells in the BM across the different groups (Figure 6E). Additionally, the composition of the cell populations was similar, including hCD34^+^hCD38^+/−^ (progenitors), hCD33^+^ (myeloid cell), and hCD19^+^ (B lymphocyte) populations (Figure 6F). In NSG mice transplanted with LV.IDUAsp.h*GALC*.APO- and LV.h*GALC*-transduced cells, we evaluated GALC activity in the spleen and liver, representing the early colonization targets of transplanted cells, and in CNS and PNS tissues, critical therapeutic targets in the disease. We observed a marked increase in GALC activity in the spleen of GT-treated mice (Figure 6G). By normalizing the enzymatic activity to the percentage of spleen engraftment (percentage of CD45^+^ cells), we highlighted a superior contribution of IDUAsp.hGALC.APO to GALC activity in this organ (Figure 6G). We observed a similar advantage when we normalized GALC activity to the percentage of PB engraftment at 12 weeks, both in the spleen (Figure 6H) and the liver (Figure 6I). These results suggest that the chimeric enzyme reaches the target tissues more efficiently than the unmodified counterpart, enhancing GALC bioavailability. The engraftment of donor cells in the CNS is minimal in this experimental setting due to the sublethal conditioning regimen (A.R., data not shown). By normalizing GALC activity in the CNS tissues of transplanted mice to the percentage of PB engraftment at 12 weeks, we highlighted the advantage of the IDUAsp.hGALC.APO compared to the native counterpart (Figure 6J). This result suggests that the circulating chimeric enzyme can cross the BBB.

The chimeric enzyme is more available in the serum, spleen, and liver tissues than the native GALC. The indirect evidence suggesting the ability to cross the BBB further emphasizes its superior biodistribution *in vivo*. These promising results support further investigation in advanced pre-clinical settings.

## Discussion

We have engineered chimeric murine and human GALC enzymes with enhanced production, secretion, BBB penetration, and cross-correction capacity. Our modifications enhance enzyme performance in murine and human *in vitro* disease models and *in vivo* HSPC-GT settings.

Previous studies have explored chimeric GALC enzymes with modifications like sp changes and APO-derived binding domains. While these studies, mostly in human cell lines or fibroblasts, showed mixed results *in vivo*, the safety and efficacy in HSPC-GT settings remain underexplored.^43^^,^^44^^,^^45^ In our earlier work, we found that replacing GALCsp with IDSsp increased GALC production and secretion in neural progenitors derived from TWI mice,^40^ suggesting that this strategy has therapeutic potential. However, the benefits were less pronounced in HSPCs, the key cells in HSPC-GT, highlighting cell-specific differences in GALC production and secretion^39^^,^^58^^,^^59^ and supporting the need for further optimization in these cells. We focused on the IDUAsp for its favorable biophysical properties, which are expected to enhance GALC synthesis and secretion.^46^^,^^47^^,^^48^^,^^49^

In HSPC-GT, reliance on enzyme transport across the BBB is reduced since the engrafted myeloid cells are the primary source of functional enzyme. However, previous studies in MPSII mice showed that HSPCs engineered to express the chimeric IDS enzyme (IDS.ApoEII) achieved better CNS pathology rescue compared to unmodified IDS^37^^,^^42^ due to increased enzymatic activity and improved transcytosis across brain endothelial cells, supporting clinical translation (NCT05665166). Our study confirms that while the APO modification did not impact GALC production and secretion in neural and hematopoietic cells,^40^ it did enhance the transendothelial transfer of the chimeric enzyme in a brain endothelial cell model. The IDUAsp.mGALC.APO enzyme provided superior cross-correction of TWI neurons and glia compared to native and IDSsp-modified GALC enzymes, indicating its potential to cross the BBB and boost GALC supply in the brain. Additionally, APO may enhance GALC uptake in neural cells via LDLr,^40^ as shown for other lysosomal enzymes,^37^^,^^41^^,^^52^ potentially improving therapeutic outcomes.

The IDUAsp.mGALC.APO variant achieved significantly higher GALC enzyme production, reaching a 7-fold increase over the physiological level, compared to the 1.5- to 3-fold increase seen with similar transduction efficacy in previous studies.^31^^,^^39^^,^^40^ This variant also outperformed both native mGALC and the IDSsp.mGALC.APO in terms of expression and release, as indicated by mCherry concentration in the sup and pellets, and higher enzymatic activity after correcting for VCN. While this level of GALC activity in donor cells is not as extreme as the 50- to 100-fold increase observed with other lysosomal enzymes like ARSA or IDUA,^23^^,^^36^^,^^38^ it still holds promise for treating GALC deficiency if combined with robust donor chimerism and effective enzyme delivery. The GALC precursor protein was efficiently secreted by HSPCs and myeloid progeny and taken up by TWI neurons and glia, providing complete rescue of GALC activity (compared to partial rescue mediated by the native mGALC and the IDSsp.mGALC.APO variant). Excessive GalCer storage in TWI neural cultures can be effectively reduced with minimal GALC levels, such as those in the sup of WT cells.^40^ This limitation of the *in vitro* model restricts our ability to detect differences among constructs regarding their cross-correction capacity through residual substrate detection. The complete clearance of GalCer in TWI neuronal and glial cell cultures suggests that the chimeric enzyme operates within the lysosomes of the acceptor cells. Furthermore, the restoration of enzymatic activity and the increased GALC-mCherry content in XC cells, with equal amounts of GALC-mCherry precursor in the sup strongly support the effectiveness of the IDUAsp.m*Galc*.APO construct. This highlights the potential of IDUAsp.mGALC.APO to drive sustained myeloid-mediated cross-correction, thereby contributing to therapeutic benefits in HSPC-GT, in addition to the known neuroprotective and immunomodulatory effects.^60^

Psychosine accumulation and neuroinflammation begin in neonatal TWI mice^53^^,^^54^ and worsen over time, leading to symptom deterioration and death by PND35–40, with an average lifespan of 39 days in our TWI colony. To improve therapeutic outcomes, we performed HSPC transplantation at PND2, which is earlier than in previous studies that used PND7.^31^^,^^54^ Additionally, we used BUS as a myeloablative agent known to enhance donor brain cell engraftment in pre-clinical models,^24^ as well as in clinical HSPC-GT for LSDs.^25^^,^^61^ While the timing of CNS engraftment after TBI and BUS has been extensively investigated in adult settings,^24^^,^^62^^,^^63^^,^^64^ fewer studies explore early postnatal (PND7–10) transplantation in GLD models using TBI^31^^,^^32^^,^^54^ or BUS.^65^^,^^66^^,^^67^ We adapted a neonatal BUS conditioning protocol^68^ and found that BUS-conditioned mice showed improved survival and GALC activity rescue compared to TBI-conditioned mice. Hematopoietic cell engraftment was similar in WT and TWI mice. However, the engraftment of Lin^−^ cells was lower than that of WT tBM cells, which aligns with prior findings indicating that committed progenitors engraft faster than stem cells.^69^^,^^70^ Although HSPC engraftment in TWI mice was lower than reported in previous studies,^31^^,^^32^ increasing BUS dose or irradiation was not feasible due to high neonatal mortality. Other compounds that can enhance brain engraftment have been identified, such as pexidartinib (PLX), a small molecule that inhibits the colony-stimulating factor 1 receptor.^63^^,^^71^^,^^72^^,^^73^ However, there is a lack of data regarding the feasibility, optimal dosage, and effects of PLX use in neonatal mice. Despite the low engraftment, BUS-conditioned TWI mice showed significant levels of GALC reconstitution, reaching close to normal levels in peripheral organs and PNS, and approximately 30%–40% of normal levels in CNS tissues. These levels are comparable to those reported in prior studies using tBM-T or HSPC-GT, in which GALC reconstitution was achieved with significantly higher donor chimerism (80%–90%).^31^^,^^32^^,^^54^ These findings highlight the efficacy of the HSPC-GT approach using a chimeric GALC enzyme, which can achieve therapeutically relevant levels of enzyme activity, even in the context of limited engraftment.

Our study demonstrates that the chimeric GALC secreted by donor-derived HSPC progeny can effectively cross-correct GALC-deficient CNS cells *in vitro*, both in murine and human contexts. Moreover, we provide qualitative *in vivo* evidence of donor-derived transgenic GALC protein in the neurons and glial cells of TWI mice following HSPC-GT. Quantitative assessment of cross-correction within the CNS *in vivo* remains technically challenging, particularly in the context of low donor cell chimerism in brain tissues. To address this limitation, we employed an allogeneic transplant model in TWI mice using tBM to achieve high donor chimerism in both PB and CNS compartments. In this setting, we observed that GALC enzymatic activity was restored to approximately 30% of normal levels in freshly isolated CD45^−^ brain cells (putative endogenous brain cells), providing strong evidence that myeloid-to-neural cross-correction occurs *in vivo* and may contribute to enzyme reconstitution in recipient GALC-deficient brain cells. These findings offer a critical perspective, especially in light of previous reports suggesting limited or negligible cross-correction *in vivo* in GLD models.^74^ While differences in experimental models, transgene constructs, or assessment techniques may explain some discrepancies across studies, our results support the notion that cross-correction is a relevant mechanism contributing to the therapeutic benefit of HSPC-T and HSPC-GT in LSDs. Further studies will be essential to validate these findings, optimize cross-correction efficiency in the HSPC-GT setting, and elucidate the cellular and molecular pathways involved in GALC uptake, trafficking, and activity within different CNS cell populations.

The limited therapeutic improvements in TWI mice undergoing HSPC-GT highlight key study constraints. Effective restoration of GALC activity, a sensitive marker for assessing therapeutic benefits, relies on proper engraftment of donor HSPCs. The TWI model’s aggressive neurological progression necessitates neonatal intervention, yet the BUS conditioning protocol poses toxicity risks, leading to inadequate myeloablation and modest engraftment. This, along with the slow microglial and macrophage replacement in the CNS following HSPC-GT, limits enzymatic rescue and the neuroprotective functions of donor cells. When combined with accelerated disease progression, these factors likely explain the limited therapeutic benefits and the lack of advantage of the chimeric enzyme over native GALC. Importantly, these challenges are specific to the TWI model and do not apply to neonatal GLD patients.^6^^,^^7^ Thus, while these murine model limitations are acknowledged, they do not undermine the potential of the chimeric construct, warranting further investigation. Future studies in GLD murine models with slower disease progression^75^ will evaluate the efficacy of the chimeric GALC enzyme in enhancing treatment benefit in HSPC-GT and, potentially, in *in vivo* GT approaches targeting the CNS.

Despite similarities between human and murine GALC enzymes, protein folding, stability, and catalytic efficiency differences may influence their functional performance *in vivo*.^35^ Our study observed reduced expression levels of human GALC compared to murine GALC when expressed in murine HSPCs (Figure S5I). This discrepancy may be attributed to species-specific factors such as mRNA stability or differences in post-translational processing.^59^ These data highlight the importance of performing human-specific studies to accurately evaluate the therapeutic potential and expression profile of human GALC, particularly in translational and preclinical settings. By utilizing an optimized LV backbone^25^ and *GALC* codon optimization,^32^ we developed an LV.IDUAsp.h*GALC*.APO analogous to the murine version. When coupled with CsH as a transduction enhancer,^55^ we achieved a 15-fold increase over normal GALC activity in human HD-derived HSPCs. This increase exceeds the maximum 3-fold enhancement reported previously.^32^ The chimeric hGALC enzyme demonstrated superior expression and activity compared to the native hGALC and the IDSsp.hGALC.APO variant in human HSPCs. Additionally, it was more effective in reducing psychosine storage in GLD human neurons and glial cells. The successful production and secretion of IDUAsp.hGALC.APO by patient-derived macrophages and its effective cross-correction of GLD neurons and glial cells confirms its potential for therapeutic application in a pathological context. Considering the inherent high variability when using primary cells, data from only two patients limit our ability to establish the superiority of the chimeric construct in this specific setting. Still, the rarity of these samples restricted our capacity to perform more replicates. Despite these challenges, we confirmed that both vectors could effectively transduce GLD samples, providing PoC for the functionality of the *GALC* constructs in these cell types and confirming the cross-correction ability of the secreted chimeric GALC.

Our xenotransplantation studies using NSG mice—an established model for assessing the efficacy of HSPC-GT and long-term gene marking in the human context—demonstrated the efficient engraftment of transduced HSPCs. In this system, HSPCs engineered to express the chimeric GALC enzyme led to increased GALC activity in serum and spleen compared to control groups, despite similar or lower engraftment levels. As expected, donor cell engraftment in other tissues, such as the liver and brain, was limited, reflecting a well-recognized limitation of the NSG xenotransplant model.^76^ This constraint made detecting significant increases in GALC enzymatic activity in these organs challenging. However, when GALC activity was normalized to the percentage of donor cells in PB, a relative enrichment was observed in the spleen, liver, and brain, suggesting that the chimeric GALC enzyme exhibits improved bioavailability and tissue penetration. This finding suggests that improved enzyme systemic bioavailability could reduce vector dosage and enhance the therapy’s safety. Overall, these *in vitro* and *in vivo* results underscore the importance of myeloid-mediated cross-correction in CNS cells as a key mechanism contributing to the therapeutic efficacy of HSPC-GT in LSDs. Additionally, they suggest that the APO modification enhances GALC delivery to the brain and reduces storage, similar to previous findings reported for IDS^37^ and ARSA enzymes.^52^

In summary, our findings underscore the potential of the IDUAsp.GALC.APO variant, combined with optimized HSPC transplantation methods, to significantly enhance therapeutic outcomes of HSPC-GT for GLD. This construct enables robust enzyme production, efficient secretion, and widespread tissue bioavailability, ultimately facilitating enzymatic correction across all affected organs and systems.

## Materials and methods

### LVs production and titration

The m*Galc* plasmid was obtained by inserting the mCherry sequence (mCherry monomeric derivative of dsRed fluorescent protein; sequence author: Clontech [TaKaRa]; https://www.snapgene.com/resources/plasmidfiles/?set=fluorescent_protein_genes_and_plasmids&plasmid=mCherry) downstream of the murine *Galc* cDNA^51^ using the aminoacidic linker TRTRPLE.^40^ The chimeric IDSsp.mGALC.APO enzyme was obtained from the m*Galc* as previously described.^40^

The chimeric IDUAsp.mGALC.APO enzyme was obtained from the m*Galc* by (1) replacing the GALCsp (MANSQPKASQQRQAKVMTAAAGSASRVAVPLLLCALLVPGGA) with the IDUAsp (IMRPLRPRAALLALLASLLAAPPVAPAE) and (2) adding a tandem repeat of the ApoE II receptor-binding region (APO), from amino acids 141–149 (APO: LRKLRKRLL LRKLRKRLL) downstream of the mCherry sequence using a flexible linker (LGGGGSGGGGSGGGGSGGGGS), as described.^37^

The chimeric hGALC enzymes were obtained from the codon-optimized human *GALC* (h*GALC*) sequence^32^ by (1) replacing the GALCsp (MTAAAGSAGRAAVPLLLCALLAPGGA) with the IDSsp (MPPPRTGRGLLWLGLVLSSVCVALG) or IDUAsp (MRPLRPRAALLALLASLLAAPPVAPAE) and (2) adding the APO sequence, as described above for the murine enzyme.

The plasmids coding for murine (IDUAsp.m*Galc*.APO) and human (h*GALC*, IDSsp.h*GALC*.APO, and IDUAsp.h*GALC*.APO) GALC enzymes were synthesized by Gene Script (Piscataway, NJ). The m*Galc* and IDSsp.m*Galc*.APO constructs used for *in vitro* and *in vivo* experiments have been described previously.^40^ Transgene expression was driven by the hPGK promoter. For the human construct, we utilized the lentiviral backbone from the HSPC-GT clinical trial for MPSI,^25^ replacing the IDUA sequence with codon-optimized native and chimeric h*GALC* sequences. The substitutions were performed using EcoRI-HF and SalI-HF restriction enzymes (New England Biolabs, Ipswich, MA) following the manufacturer’s instructions. The LV expressing GFP under the hPGK promoter (LV.GFP) was used as a control.^77^ VSV-pseudotyped third-generation LVs were produced by transient four-plasmid co-transfection into HEK293T cells and purified by ultracentrifugation, as described.^78^^,^^79^ Expression titers and infectivity of vectors were assessed by quantitative droplet digital PCR (ddPCR) as previously described^80^ and reported in Table S2.

### Cell isolation, culturing, and treatment

Cells were maintained in a 5% CO_2_ humidified atmosphere at 37°C. Cells were transduced at the indicated MOI as calculated by titration of vector batches on HEK293T cells and expressed as transducing units per HEK293T cell.^80^

#### Isolation and LV transduction of murine HSPCs

Murine HSPCs were purified from the BM of TWI and WT adult mice (30–40 days) by Lin^−^ selection using the mouse Lineage Cell Depletion Kit (Miltenyi Biotec, Bergisch Gladbach, Germany) according to the manufacturer’s instructions. Cells were plated and transduced with LVs (MOI 100 for 12 h) as described.^40^ After 10 days, we counted the number of colonies (CFC assay) and collected the bulk pellets for VCN analysis. After 14 days of culture, the LC pellet and sup were collected for VCN, enzymatic activity, WB, IF, and ImageStream analyses. LC sup were collected for cross-correction experiments.

#### Differentiation of murine NPCs into neurons/glia and cross-correction experiments

We established independent NPC lines from TWI and WT mice as previously described.^81^^,^^82^ Serially passaged neurospheres were dissociated, and single cells were plated (4E+4 cells/cm^2^) onto Matrigel (Corning, Bedford, MA)-coated wells in complete medium, as described by Ricca et al.^82^ After 2 days, we exposed them to fibroblast growth factor 2 (FGF2, Tebubio, Île-de-France, France)-containing medium (48 h) and then to a mitogen-free medium added with fetal bovine serum (FBS; EuroClone, Milan, Italy) for 5 days, to promote neuronal and glial differentiation.^40^ In the last 3 days of differentiation, TWI neuronal/glial cells were exposed every 24 h to the sup of donor cells, namely UT WT HSPCs or LV-transduced TWI HSPCs (LC). XC cells, UT WT, and TWI controls were collected for GALC intracellular enzymatic activity and ELISA analyses.

#### Murine bEND.3 cells

bEND.3 cells (immortalized brain endothelioma murine cell line) were cultured in Dulbecco’s modified Eagle’s medium (high glucose; Sigma-Aldrich, St. Louis, MO), supplemented with 10% FBS, 1% penicillin/streptomycin (P/S; Lonza, Basel, Switzerland), and 1% glutamine (Sigma-Aldrich), at a density of 5E+5 cells/cm^2^. Adherent cells were detached using 0.25% trypsin-4 mM ethylenediaminetetraacetic acid solution (Thermo Fisher Scientific, Waltham, MA). The cell culture medium was replaced every 2 days. bEND.3 cells were plated onto 150-μg/mL collagen-coated transwell membranes (12-mm Ø inserts, pore size 3.0 μm, growth area 1.12 cm^2^; COSTAR, Corning, Tewksbury, MA) as described^83^ for the analyses of GALC transcytosis. Permeability studies to assess the optimal conditions ensuring cell confluency were performed using 4 kDa fluorescein isothiocyanate-dextran (200 μg/mL, Sigma-Aldrich; 30 min of incubation at 37°C) as described.^83^ The fluorescence of the liberated molecule was measured with a spectrofluorometer (λ excitation 485 nm, λ emission 535 nm).

#### Human CD34^+^ HSPC transduction

BM or granulocyte-colony-stimulating factor mPB human CD34^+^ cells (clinical protocol: Tiget05) were purchased from Lonza and plated at 1E+5 cells/cm^2^ in RetroNectin (TaKaRa Bio, San Jose, CA)-treated plates in serum-free StemSpan medium (STEMCELL Technologies, Vancouver, Canada) supplemented with P/S, recombinant human stem cell factor (rhSCF), recombinant human thrombopoietin, recombinant human Flt3 ligand, and recombinant human interleukin-6 (rhIL-6) (all from PeproTech, Cranbury, NJ) 22 ± 2 h before transduction. CsH (Sigma-Aldrich) was added to LV transduction media and maintained for 14 ± 1 h, as described.^55^ HSPCs were washed, counted, and plated for the CFC assay (3E+2–4E+2 cells/mL in human MethoCult; STEMCELL Technologies) or to obtain liquid cultures in Iscove’s modified Dulbecco’s medium (Corning) supplemented with P/S, human cytokine (rhSCF by Miltenyi Biotec, rhIL3 and rhIL6 by PeproTech), and 10% FBS (LC medium). LCs were counted every 2 days and plated at 1E+5 cells/cm^2^ in the LC medium. After 14 days of culture, pellets and sup were collected for VCN, enzymatic activity, and WB analyses. After 14 days, CFCs were counted, and the bulk pellets were collected for VCN and fluorescence-activated cell sorting (FACS) analyses. Sup from LC was collected for cross-correction experiments.

#### Human CD14^+^-derived macrophages

Human CD14^+^ monocytes were purified from PB mononuclear cells of HD (*n* = 6) and a late infantile GLD patient (*n* = 2) using CD14 MicroBeads (Miltenyi Biotec), according to the manufacturer’s instructions. To obtain macrophages, monocytes were plated in RPMI 1640 (Thermo Fisher Scientific) supplemented with FBS, P/S, human serum (EuroClone), and l-glutamine (Sigma-Aldrich) in the presence of human recombinant macrophage-CSF (M-CSF, Miltenyi Biotec), as previously described.^56^ For transduction, monocytes were incubated for 6 h with the accessory viral protein vpl-VPX (4 μL/1E+6 cells),^84^ followed by overnight transduction at the MOI of 5.. Viral-containing sup was removed, and cells were incubated with a growth medium for 9 days (M0 phenotype). Pellet and sup were collected for VCN and GALC enzymatic activity analyses. Sup from macrophages was collected for cross-correction experiments.

#### Human iPSCs and neuronal/glial differentiation

We induced neural differentiation of HD and GLD hiPSCs using a dual-Smad inhibition method.^85^ iPSC colonies were detached with ACCUTASE (Sigma-Aldrich) and plated as single cells on Matrigel-coated dishes in StemMACS iPS-Brew XF medium (Miltenyi Biotec) with ROCK inhibitor Y-27632 (Sigma-Aldrich). After reaching 90% confluence, we switched to knockout serum replacement medium (Invitrogen, Waltham, MA) with Noggin (R&D Systems, Minneapolis, MN) and SB431542 (Sigma-Aldrich) for 4 days, followed by a gradual transition to N2 medium with Noggin and SB431542. Human iPSC-derived-neural stem/progenitor cells (NPCs) were expanded in N2 medium with basic FGF, epidermal growth factor (PeproTech), and ROCK inhibitor (Sigma-Aldrich). NPCs at passages 2–3 were detached and plated on Matrigel-coated dishes in the same medium for differentiation. N2 medium was gradually replaced with glial differentiation medium containing platelet-derived growth factor-AA, neurotrophin-3, insulin growth factor-1, hepatocyte growth factor (PeproTech), and triiodothyronine (Sigma-Aldrich). From day 14 onward, cells were maintained in glial maturation medium with ascorbic acid, excluding growth factors, as described.^57^^,^^85^ Human iPSCs, hiPSC-derived NPCs, and differentiated progeny were maintained in a humidified atmosphere with 5% O_2_ and 5% CO_2_ at 37°C. In the last 24 h of differentiation, GLD differentiated cells were exposed to the sup of donor cells (UT HD or LV-transduced HD CD34^+^ progeny; UT HD, GLD, or LV-transduced macrophages). XC cells and UT HD and GLD controls were analyzed for GALC intracellular enzymatic activity assays and dosage of psychosine (by mass spectrometry, service outsourced to the Laboratory for Genetic Metabolic Diseases, Academic Medical Center, University of Amsterdam, Amsterdam, the Netherlands).

### Analyses of GALC transcytosis using bEND.3 cells in a transwell system

bEND.3 cells were plated onto collagen-coated transwell membranes as described previously. After quantifying mGALC and IDUAsp.mGALC.APO in the donor sup (by mCherry ELISA; Abcam, Cambridge, UK), an equal amount of GALC-enriched medium was used in the subsequent experiments. PBS was added to the lower chamber of the transwell, while the GALC-enriched medium was added to the upper chamber. Transcytosis of GALC to the basolateral chamber was assessed by mCherry ELISA after 24 h of incubation in a 5% CO_2_ humidified atmosphere at 37°C.

### Quantification of VCN

We isolated genomic DNA from cellular pellets and pellets from the BM of treated mice using the Qiagen mini or micro kit (Qiagen, Hilden, Germany) following the provided instructions. DNA was quantified using the NanoDrop ND-1000 Spectrophotometer by measuring the optical density at 260/280 nm. The VCN was assessed using quantitative ddPCR, following the method outlined by Ornaghi et al.^80^

### Total mRNA extraction and reverse transcription-PCR

Following the instructions, we extracted total RNA from cellular pellets using the RNeasy mini or micro kit (Qiagen). RNA quantification was performed with the NanoDrop ND-1000 Spectrophotometer. Following the manufacturer’s protocols, reverse transcription was conducted with 1 μg total RNA and the QuantiTect Reverse Transcription Kit (Qiagen).

qPCR was performed as previously described.^40^ The probe and primers (TaqMan Gene Expression Assays, Applied Biosystems, Waltham, MA) are listed below:

Endogenous human *GALC*: Hs01012300_m1
Exogenous h*GALC* forward: 5′-GCGGAAGATGCTGAACTACC-3′
Exogenous h*GALC* reverse: 5′-GTGAAGTACTCGAACACGCC-3′

### GALC activity assay

GALC activity was assessed in cells, culture media, serum, and tissues as described.^86^

### IF

IF analysis was conducted on cultured murine cells, human cells, and mouse tissues following established protocols.^40^^,^^87^ Primary and secondary antibodies utilized are detailed in Table S3. Confocal images were acquired at 4×, 20×, 40×, or 63× magnification using a Leica TCS SP8 confocal microscope (Leica, Wetzlar, Germany) or the Mavig RS-G4 confocal microscope (MAVIG Research, Munich, Germany) and analyzed using LasX (Leica Application Suite X, RRID: SCR_013673) or Imaris (Oxford Instruments, Abingdon-on-Thames, UK) software, respectively. Images were imported into ImageJ or Adobe Photoshop 2021 to adjust brightness, contrast, and merge channels.

### WB

Cell pellets and tissues were resuspended in 50–200 μL (for cells) or 500 μL (for tissues) of radioimmunoprecipitation assay lysis buffer enriched with protease (cOmplete Tablets, Roche, Basel, Switzerland) and phosphatase (PhosSTOP, Roche) inhibitors. Tissues underwent lysis using a homogenizer. Protein extraction was performed as previously described.^40^ Protein concentration was determined using the DC Protein Assay (Bio-Rad, Hercules, CA) and the Multiskan Go Microplate Spectrophotometer (Thermo Fisher Scientific). Proteins from the sup of cultures plated at the same cell density were obtained as previously described.^40^ SDS-PAGE was employed to fractionate 5–30 μg protein from the cell pellet and sup using NuPAGE 4%–12% BisTris Protein Gels (Invitrogen), followed by transfer to nitrocellulose or polyvinylidene difluoride (PVDF) membranes (Invitrogen) using the iBlot2 Gel Transfer Device (Invitrogen). Proteins were transferred to PVDF membranes (Millipore, Burlington, MA) for 2 h at 400 mA to detect native and chimeric hGALC. We used the protein marker PM 2610 (SMOBiO, Paramount, CA). Immunodetection was performed using Clarity ECL Western Blotting Substrate (Bio-Rad) and imaged with the Alliance Western Blot Imaging System (UVItec, Cambridge, UK). Quantification of WB was conducted using ImageJ software, following the guidelines outlined in section 30.13 of the ImageJ User Guide version 1.46.

### ImageStream

LV.IDUAsp.m*Galc*.APO-transduced TWI HSPCs (LC; 3E+6 cells) were stained and analyzed as previously described.^40^ At least 2E+5 events were collected at 60× magnification, and approximately 8E+4 cells were analyzed. The presence or absence of at least one overlapping mask (proximity or co-localization mask) was quantified.

### ELISA for mCherry detection

Following instructions, quantitative measurement of mCherry protein in cell culture sup and cell extract samples was performed through the mCherry SimpleStep ELISA kit (Abcam). Neuronal/glial cells were lysed directly into the well in 70 μL 1× cell extraction buffer PTR. Lysates were collected, incubated on ice for 15 min, and centrifuged at 16,000 × *g* at 4°C for 15 min. Protein concentration was determined using the DC Protein Assay (Bio-Rad) and the Multiskan Go Microplate Spectrophotometer (Thermo Fisher Scientific). We used 3 μg protein for mCherry detection. Sup were diluted to allow measurability and adjusted to equal amount for the different experimental conditions.

### Psychosine dosage

Galactosylsphingosine (LysoGalCer; psychosine) was quantified in treated and UT neural cells derived from iPSCs from patients with GLD and HD as controls. The analytical procedure was adapted from previously described protocols for glycosphingolipid and lysosphingolipid quantification in plasma and serum.^88^^,^^89^^,^^90^ Cell pellets were homogenized in 150 μL water by sonication on ice. A 50-μL aliquot was taken to determine protein concentration using the DC Protein Assay. For lipid extraction, 75 μL homogenate was mixed with internal standards of 25 pmol LysoGalCer-d7 (25 μL, 1 μM in methanol). Subsequently, 240 μL methanol and 150 μL chloroform were added. After vortexing and incubation at room temperature, samples were centrifuged at 15,700 × *g* for 10 min at 4°C to precipitate protein. The sup was transferred to a 2-mL tube, and 150 μL chloroform and ammonium formate/formic acid buffer (225 μL, pH 3.15) were added. After vortexing, samples were centrifuged at 15,700 × *g* for 3 min at 4°C to separate phases, and the upper phase was dried under nitrogen at 40°C. After butanol/water partitioning, the upper phase was again dried and reconstituted in 100 μL mobile phase B (95:5 acetonitrile:water with 1 mM ammonium formate and 0.1% formic acid). The lower phase (glycosphingolipids) was dried under nitrogen and subjected to deacylation by incubation with 0.5 mL 0.1 M NaOH in methanol using a microwave program. After neutralization with 50 μL 0.1 M HCl in methanol, the processed samples were combined with the lysosphingolipid workflow from the drying step. ultra-performance liquid chromatography (UPLC)-tandem mass spectrometry analysis was performed on a Waters Acquity UPLC system coupled to a Waters Xevo TQ-XS mass spectrometer, operating in positive electrospray ionization mode. Chromatographic separation was achieved on an Ascentis Express HILIC column (4.6 × 50 mm, 2.7 μm, Supelco, Bellefonte, PA) with an HILIC SecurityGuard precolumn (4 × 3.0 mm, Phenomenex, Torrance, CA) at room temperature. The mobile phases consisted of (A) water with 1 mM ammonium formate and 0.1% formic acid and (B) acetonitrile:water (95:5) with 1 mM ammonium formate and 0.1% formic acid. A gradient elution was applied at 1.5 mL/min with the following program: 0–0.2 min, 100%–95% B; 0.2–3.5 min, 95% B; 3.5–4.0 min, 95%–10% B; 4.0–5.0 min, 10% B; 5.0–5.1 min, 10%–100% B; and 5.1–7.0 min, 100% B. The injection volume was 10 μL. Analytes of specific mass transitions were detected using multiple reaction monitoring. Quantification was performed against matrix-matched calibration curves using deuterated internal standards. Data acquisition and processing were carried out using MassLynx software. Psychosine concentration was normalized to the protein content of the homogenate and expressed as pmol/g of protein.

### Mice

NOD.Cg-PrkdcscidIL2rgtm1Wjl/SzJ (NSG), transgenic CAG-GFP (TgCAG-GFP), background C57BL/6-Tg (CAGeGFP1Osb/J), and TWI mice were purchased from The Jackson Laboratory (Bar Harbor, ME). Mouse colonies were maintained in the animal facility of the San Raffaele Scientific Institute, Milano, Italy.

### *In vivo* treatments

#### Myeloablative regimen

Between 16 and 24 h before transplantation, neonatal (PND1–2) TWI and WT pups of both sexes underwent conditioning either with sublethal TBI at 400 cGy or a single intraperitoneal injection of 20 mg/kg BUS (Busilvex, 6 mg/mL, Pierre Fabre, Boulogne-Billancourt, France). UT, TWI, and WT littermates were utilized as the control group. Female NSG mice aged 8–10 weeks were conditioned with sublethal TBI at 180 cGy as described.^55^ Conditioned mice were given gentamycin (final concentration 320 mg/mL, Italfarmaco, Milan, Italy) in their drinking water starting from the day of conditioning for a subsequent 2 months.

#### Total BM transplant

We used 4- to 8-week-old TgCAG-GFP mice as donors and euthanized them using CO_2_. BM from their tibias and femurs was flushed out with PBS and centrifuged at 500 × *g* for 5 min. Red blood cells in the BM pellet were lysed with double-distilled water for 10 s, and the reaction was stopped by adding PBS with 10% FBS. The cell suspension was then filtered through a 40-μm cell strainer (BD Biosciences, Franklin Lakes, NJ) and centrifuged at 500 × *g* for 5 min. The cells were resuspended in PBS (5E+6 cells/50 μL) and immediately injected into myeloablated recipient mice. The donor BM cells expressed GFP and had physiological GALC activity levels. Neonatal recipient mice (PND2–3) were briefly anesthetized with ice for 1 min to induce transient hypothermia. The donor cells were injected into the temporal vein using a U-100 insulin syringe (DB Micro-Fine, 0.3 mL). After injection, the pups were warmed under a heat lamp for approximately 1 min. The entire procedure took less than 3 min per mouse, after which the neonates were promptly returned to their parental cages. The survival rate after the procedure exceeded 95%. Experimental animals of both sexes were randomly assigned to groups before determining their sex. UT TWI and WT littermates were included as controls. No differences in treatment outcomes based on sex were observed.

#### HSPC GT

HSPCs (Lin^−^ cells) from WT and TWI mice were isolated as described previously.^80^ The day after transduction with different vectors (LV.GFP at 50 MOI; LV.m*Galc* and LV.IDUAsp.m*Galc*.APO at 100 MOI), the HSPCs were suspended in PBS (5E+5 cells/50 μL) and immediately injected using a U-100 insulin syringe (DB Micro-Fine, 0.3 mL) into the temporal vein of myeloablated recipient mice, as described above. Experimental animals of both sexes were randomly assigned to groups before determining their sex. UT TWI and WT littermates were included as controls. No differences in treatment outcomes based on sex were observed.

#### Xenotransplantation of human HSPCs

CD34^+^ HSPCs derived from HD were transduced with LV.h*GALC*, LV.IDUAsp.h*GALC*.APO, and LV.GFP as control at 100 MOI. Conditioned NSG female mice were intravenously transplanted via retroorbital injection or tail vein with 3–5E+5 cells in a 100- to 150-μL PBS suspension using a U-100 insulin syringe (DB Micro-Fine, 0.3 mL).

### Tissue collection and processing

Treated and control mice were anesthetized with ketamine-xylazine (from Sigma, 100 and 10 mg/kg, respectively) and intracardially perfused via the descending aorta with 0.9% NaCl + 25,000 heparin sodium IU/mL (PharmaTex, Milan, Italy). Brain, spinal cord, sciatic nerve, liver, spleen, and BM tissues were collected for enzymatic activity. The two brain hemispheres were separated, and each hemisphere was again divided into two parts. An integral hemisphere was used for IF analysis and to freshly isolate CD45^+^ and CD45^−^ cell populations. For the other, a cut was made, thus separating the rostral region (RO), comprising the telencephalon, diencephalon, and midbrain, and the caudal region (CA), comprising the cerebellum, pons, and medulla. The RO and CA regions were analyzed for WB and pooled to analyze the GALC enzymatic activity. The spinal cord was collected as a whole and then sagittally halved. The BM was collected as described above and immediately frozen for subsequent enzymatic activity and VCN analyses. For IF analysis, sections of the brain and spinal cord tissues were fixed for 24 h in 4% paraformaldehyde (Santa Cruz Biotechnology, Dallas, TX) and included in 4% agarose (EuroClone) as previously reported.^87^ Serial coronal vibratome sections (6 series, 40 μm thick) were stained as described above. For biochemical and molecular assays, tissues were quickly frozen in liquid nitrogen.

### Adult brain dissociation

CD45^+^ myeloid cells and a mixed CD45^−^ cell population, consisting of neuronal, glial, and endothelial cells, were freshly isolated using the Adult Brain Dissociation Kit (Miltenyi Biotec) according to the manufacturer’s protocol. CD45^+^ and CD45^−^ cell fractions were subsequently analyzed for cytofluorometric and GALC enzymatic activity analyses.

### Cytofluorometric analyses

#### Cell composition of LV-transduced CD34^+^ progeny

We incubated 2E+5 cells from the LC and CFC bulk in FACS buffer (PBS, 5% FBS, 1% BSA) for 15 min. After incubation for 30 min at 4°C with antibodies (listed in Table S3), cells were centrifuged at 500 × *g* for 5 min and resuspended in FACS buffer as previously described.^91^

#### Engraftment and composition of donor-derived cells in treated mice

PB and BM were collected from treated and UT mice. The spleen collected from UT and treated NSG mice was smashed through a cell strainer (40 μm) in PBS. The CD45^+^ and CD45^−^ cell populations were freshly isolated from the brains of tBM-T and UT mice as described above. We incubated 1–2E+5 cells or 20 μL PB of each sample for 30 min at 4°C with antibodies (listed in Table S3). The GFP and mCherry signals were measured by direct fluorescence. The PB samples were incubated for 15 min on ice with 1 mL ammonium-chloride-potassium (Thermo Fisher Scientific) buffer for red blood cell lysis following the manufacturer’s instructions. Samples were centrifuged at 500 × *g* for 5 min and resuspended in FACS buffer. Cell suspensions were analyzed using a flow cytometer (Canto II, BD Biosciences; Cytoflex, Beckman Coulter, Brea, CA). Data were analyzed using FlowJo software.

### Statistical analysis

Data were analyzed with GraphPad Prism version 10.0 for Macintosh and expressed as the mean or mean ± standard deviation (SD) when *n* ≥ 2. One-way ANOVA or Kruskal-Wallis followed by appropriate post-tests and unpaired *t* test or Mann-Whitney tests were used. The correlation analysis was performed using Spearman’s correlation. Survival curves were analyzed using the log rank (Mantel-Cox) test. The *p* value threshold for statistical significance was considered to be 0.05. The number of samples and statistical tests used are indicated in the figure legends.

## Data availability

The data for this publication are available upon request to scientific community members for research purposes.

## Acknowledgments

We are grateful to Luigi Tiradani and Francesca Ornaghi for vector preparation and titration; Vasco Meneghini, Filippo Casalini and Ilaria Laface for help in CD14^+^ cell culture and differentiation; Tiziano Di Tomaso for assistance with the cloning strategy; Bernhard Gentner for providing the LV backbone; Alessandra Biffi for providing the codon-optimized h*GALC* cDNA; Janet E. Deane for providing the anti-hGALC antibody; Desirèe Zambroni for the ImageStream analysis; Valeria Berno and Cesare Covino for confocal microscopy support; Alessandro Nonis (University Centre of Statistics in Biomedical Sciences – CUSSB, Vita-Salute San Raffaele University, Milan, Italy) for support with the statistical analysis; Frèdéric M. Vaz (University of Amsterdam, The Netherlands) for psychosine analysis; and all the members of the Gritti lab for continuous support and helpful discussion. Part of this work was carried out in ALEMBIC (Advanced Light and Electron Microscopy BioImaging Center) and FRACTAL (Flow Cytometry Resource, Advanced Cytometry Technical Applications Laboratory), the core facilities established at IRCCS Ospedale San Raffaele and Vita-Salute San Raffaele University, Milan, Italy. All animal procedures were performed according to protocols approved by the Institutional Committee for the Good Animal Experimentation of the San Raffaele Scientific Institute (IACUC nos. 791, 1145, and 1192) and are reported to The Ministry of Health, as required by Italian law. Human cells were used according to the guidelines on human research issued by the ethics committee of Ospedale San Raffaele in the context of the protocols TIGET-HPCT, Tiget05 (GR-2019-12369357), and Tiget09 (GR2019-microMLD and 12368930). This study was funded by grants from 10.13039/501100002426Fondazione Telethon, Italy (no. TTAGD0222TT) to A.G.; European Leukodystrophies Association (no. ELA 2019-015I2) to A.G.; 10.13039/501100003196Italian Ministry of Health (no. GR-2019-12369357) to A.R. (principal investigator [PI]), A.K.R. (co-PI), and F.M.; and 10.13039/100031271Fondazione Centro San Raffaele (FCSR)-2019 fellowship program to F.C. The sponsor(s) had no role in the study design, data collection, analysis, and interpretation, or the decision to submit the article for publication. F.C. conducted part of this study to fulfill the requirements of his Ph.D. in Molecular Medicine, XXXV cycle (Vita-Salute San Raffaele University, Milan, Italy), with the support of fellowships co-funded by 10.13039/501100024370Ministero dell’Istruzione e del Merito (MIUR) and Vita-Salute San Raffaele University.

## Author contributions

F.C. and A.R. contributed to the conception and design of the study, wrote the manuscript, and performed the statistical analysis; F.C., A.R., I.P., M.F., and V.S. performed the *in vitro* and *in vivo* experiments; E.V. and G.U. performed the *in vitro* experiments on CD34^+^ HSPCs; S.M. supervised the biochemical analyses; F.M. performed the biochemical analyses; A.K.-R. provided expertise, resources, and intellectual input; A.G. designed and supervised the study, provided resources, wrote the manuscript, and approved the final version. All authors contributed to the manuscript revision and read and approved the submitted version.

## Declaration of interests

The authors declare no competing interests.

## Supplemental information

Document S1. Figures S1–S5 and Tables S1–S3

Document S2. Article plus supplemental information
