# Supplementary material for: Chimeric enzymes enhance treatment potential for globoid cell leukodystrophy through hematopoietic stem cell gene therapy
Source: Mol Ther. 2025 Sep 22;33(12):6226–47. doi: 10.1016/j.ymthe.2025.09.030 (PMC12703158; doi:10.1016/j.ymthe.2025.09.030)
Supplement: Document S1. Figures S1–S5 and Tables S1–S3 [file mmc1.pdf]

YMTHE, Volume *33*

Supplemental Information

Chimeric enzymes enhance treatment potential for globoid cell leukodystrophy through hematopoietic stem cell gene therapy

Federica Cascino, Alessandra Ricca, Ilaria Picciotti, Erika Valeri, Giulia Unali, Veronica Saporito, Marta Freschi, Francesco Morena, Sabata Martino, Anna Kajaste-Rudnitski, and Angela Gritti

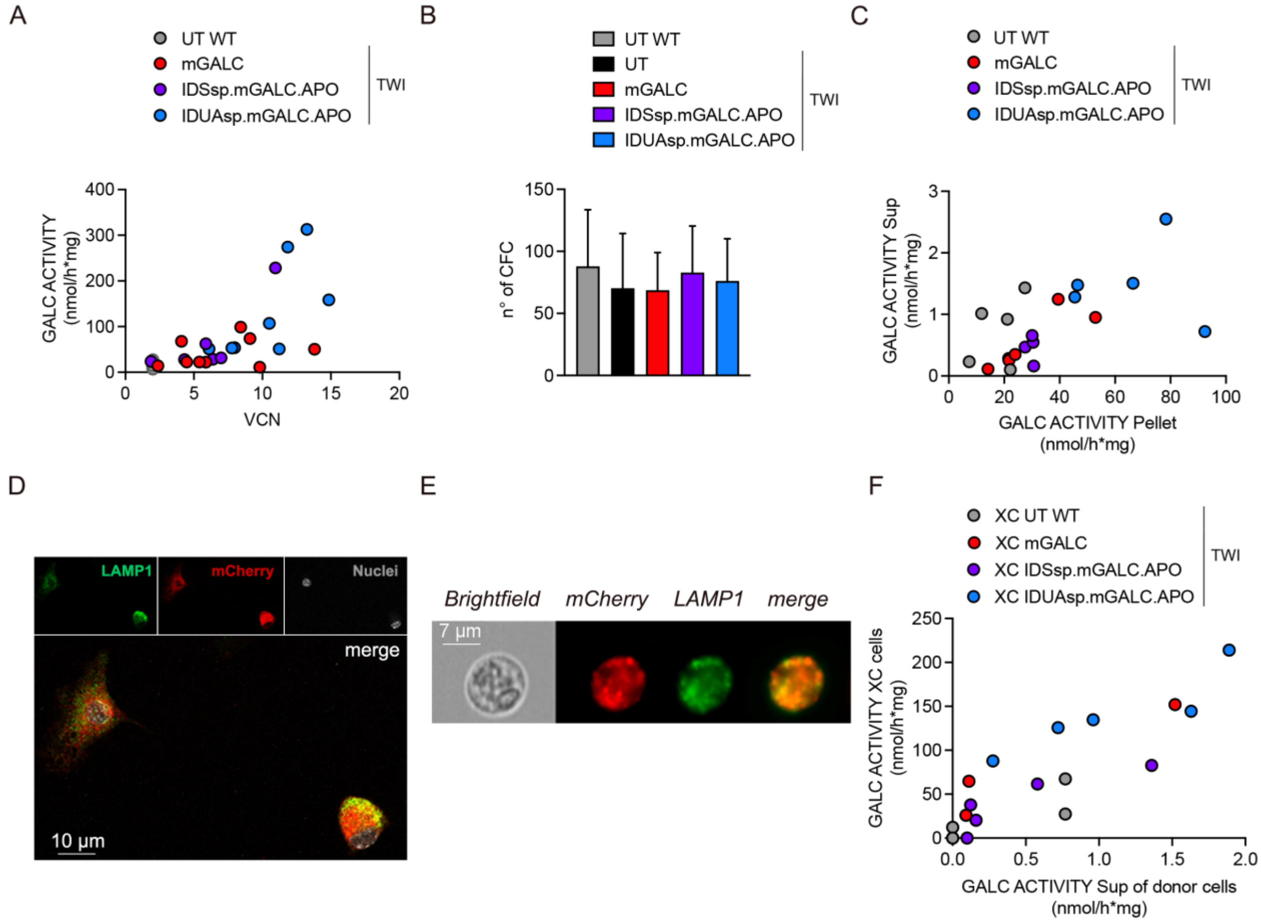

Figure S1. The LC progeny from LV-transduced TWI HSPCs overexpresses chimeric GALC enzymes.
(A) Correlation between GALC activity and VCN measured in LV-transduced TWI HSPC progeny (LC; pellets). n=6-9 experiments, 1-3 technical replicates/experiment. Spearman correlation, r = 0.7, p<0.0001. **(B)** Number of colonies (CFC assay) originated from UT and LV-transduced TWI HSPCs and UT WT counterparts. Data are expressed as the mean (SD), n=6-9 experiments, 2 technical replicates/experiment. **(C)** Correlation between GALC activity in the pellet and sup measured in LV-transduced TWI HSPC progeny (LC). n=4-5 experiments, 1-3 technical replicates/experiment. Spearman correlation, r = 0.6, p = 0.0058. **(D)** Representative confocal IF images of LC cultures from LV-transduced TWI HSPCs showing IDUAsp.mGALC.APO expression (mCherry, red) in lysosomes (LAMP1, green). Nuclei stained with Hoechst (grey, pseudo colour); 63X magnification. Scale bar: 10 μm. **(E)** Representative ImageStream pictures showing LAMP1 (green, pseudocolour) and mCherry (red, pseudocolour) proximity (merge) in LV.IDUA.mGALC.APO-transduced TWI HSPC progeny (LC); n=1 experiment. At least 20,000 events were collected at 60X magnification, and approximately 8,000 cells were analysed. Proximity index = 88%. Scale bar: 7 μm. **(F)** Correlation between GALC activity in the sup of LV-transduced HSPC progeny (donor cells) and GALC activity in XC TWI neural cells (acceptor cells). n=3-5 experiments, 2 technical replicates/experiment. Spearman correlation, r = 0.88, p<0.0001.

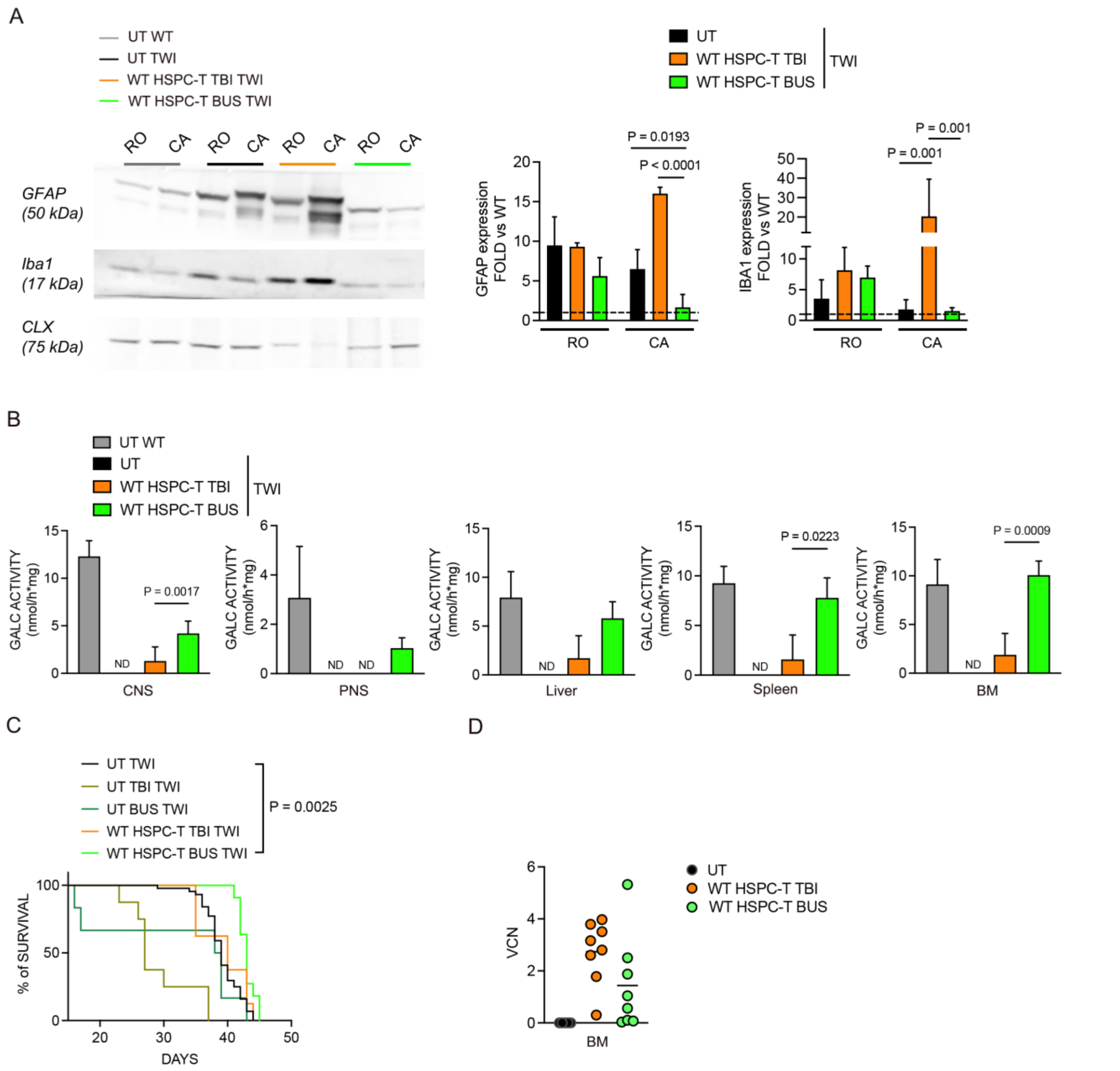

Figure S2. Busulfan as a myeloablative regimen in neonatal TWI mice.

(A) Representative WB analysis showing GFAP and Iba1 protein expression in the rostral (RO) and caudal (CA) brain regions of treated mice and UT controls (WT and TWI). Calnexin (CLX) was used as a normalizer. Graphs show the quantification of WB analyses. n=2-3 blots in n=3 different experiments, 3-5 mice/treatment. Protein expression (GFAP or Iba1/CLX) is shown as FOLD vs UT WT (dotted line); data are expressed as the mean (SD). Kruskal-Wallis and Dunn's multiple comparison test (GFAP), Ordinary One Way ANOVA, and Tukey's multiple comparison test (Iba1). The WB membrane has been cut prior to incubation with primary antibodies to enable the simultaneous detection of different proteins. **(B)** GALC enzymatic activity measured in CNS tissues (brain and spinal cord), PNS tissue (sciatic nerve), peripheral organs (liver, spleen), and bone marrow (BM) of treated mice and UT controls (WT and TWI). Data are expressed as the mean (SD); n=5-8 mice/group. Kruskal-Wallis and Dunn's multiple comparison test **(C)** Kaplan-Meier survival curves plotting the survival percentage of treated and UT TWI mice. UT TWI, n=40; WT HSPC-T TBI, n=8; WT HSPC-T BUS, n=11; TBI TWI, n=8; BUS TWI, n=2. Log Rank (Mantel-Cox) test. **(D)** VCN was measured in the BM of TWI mice transplanted with LV.GFP-transduced WT HSPCs after TBI or BUS conditioning. n=3 experiments, each dot represents one mouse.

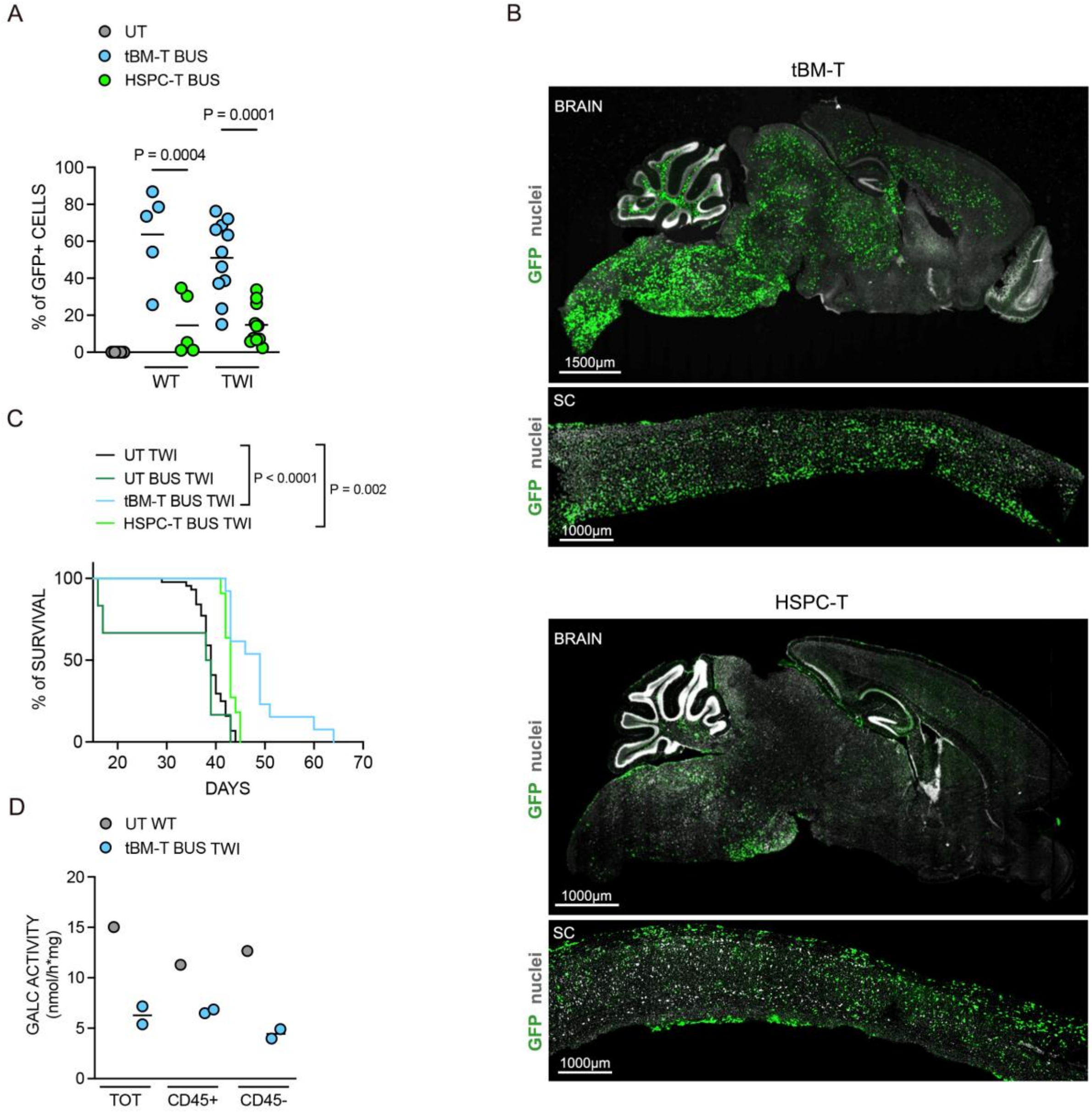

Figure S3. Comparison of HSPC myeloid progeny and tBM efficacy in neonatally-transplanted mice.
(A) Engraftment of donor-derived GFP$^+$CD45$^+$ cells measured in the peripheral blood (PB) of WT and TWI mice, evaluated one month after BUS conditioning and transplantation of WT total bone marrow (tBM-T) or LV.GFP-transduced WT HSPCs (WT HSPC-T). Untreated (UT) WT mice were used as controls. One-way ANOVA followed by Tukey's multiple comparisons test. Each dot represents a mouse. **(B)** Representative fluorescence pictures of sagittal brain slices showing engrafted GFP$^+$ cells (green; direct fluorescence) in the brain and spinal cord (SC) of tBM-T and HSPC-T TWI mice analysed at PND 46 and PND 43, respectively. Nuclei stained with Hoechst (grey, pseudocolour). 20X magnification. Scale bars: 1000 -1500 µm. **(C)** Kaplan-Meier survival curves plotting the survival percentage of treated and UT TWI mice. UT TWI, n=40; tBM-T TWI, n=13; WT HSPC-T TWI, n=11; BUS TWI, n=2. Log Rank (Mantel-Cox) test. **(D)** GALC activity was measured in the whole brain tissue lysates (TOT) and pellets of CD45$^+$ and CD45$^-$ populations freshly isolated from the brain of tBM-T TWI and UT WT control. Data are expressed as the mean. Each dot represents one mouse.

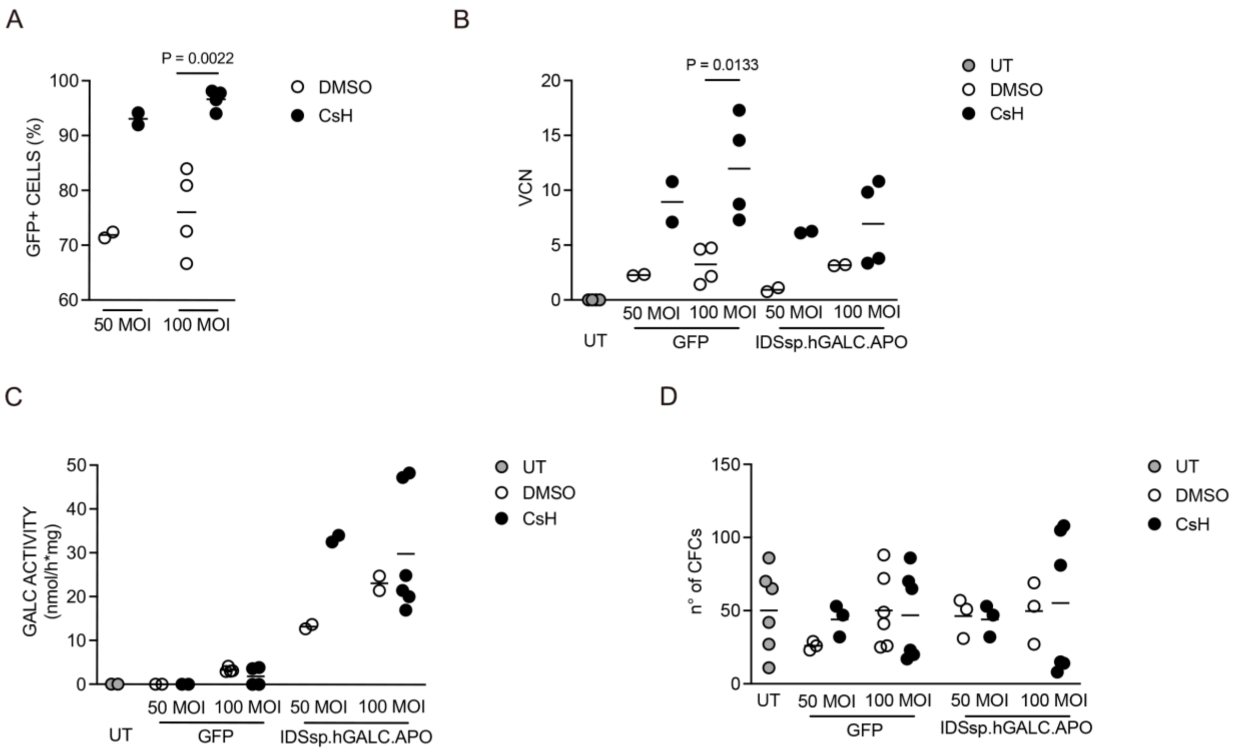

Figure S4. Cyclosporin H enhances LV transduction efficiency of human CD34⁺ HSPCs.

(A) Percentage of GFP⁺ (cytofluorimetric analysis) assessed in CD34⁺ cells transduced with LV.GFP in the presence or absence of cyclosporin H (CsH). DMSO, vehicle. Data are expressed as mean, n=1-2 experiments, 2 technical replicates/experiment. Unpaired Student's t-test. **(B)** VCN was measured 14 days post-transduction in the CD34⁺ HSPCs progeny (LC) transduced with LV.GFP and LV.IDSsp.hGALC.APO in the presence or absence of CsH. UT, untreated. Data are expressed as mean, n=1-2 experiments, 2 technical replicates/experiment. Unpaired Student's t-test. Each dot represents one replicate. **(C)** GALC enzymatic activity was measured in CD34⁺ HSPC progeny (LC) transduced with LV.IDSsp.hGALC.APO and LV.GFP, in the presence or absence of CsH. Data are expressed as mean, n=1-2 experiments, 2-4 technical replicates/experiment. Each dot represents one replicate. **(D)** Number of colonies (CFC assay) originated from LV-transduced CD34⁺ HSPCs and controls. Data are expressed as mean, n=1-2 experiments, 3 technical replicates/experiment. Each dot represents one replicate.

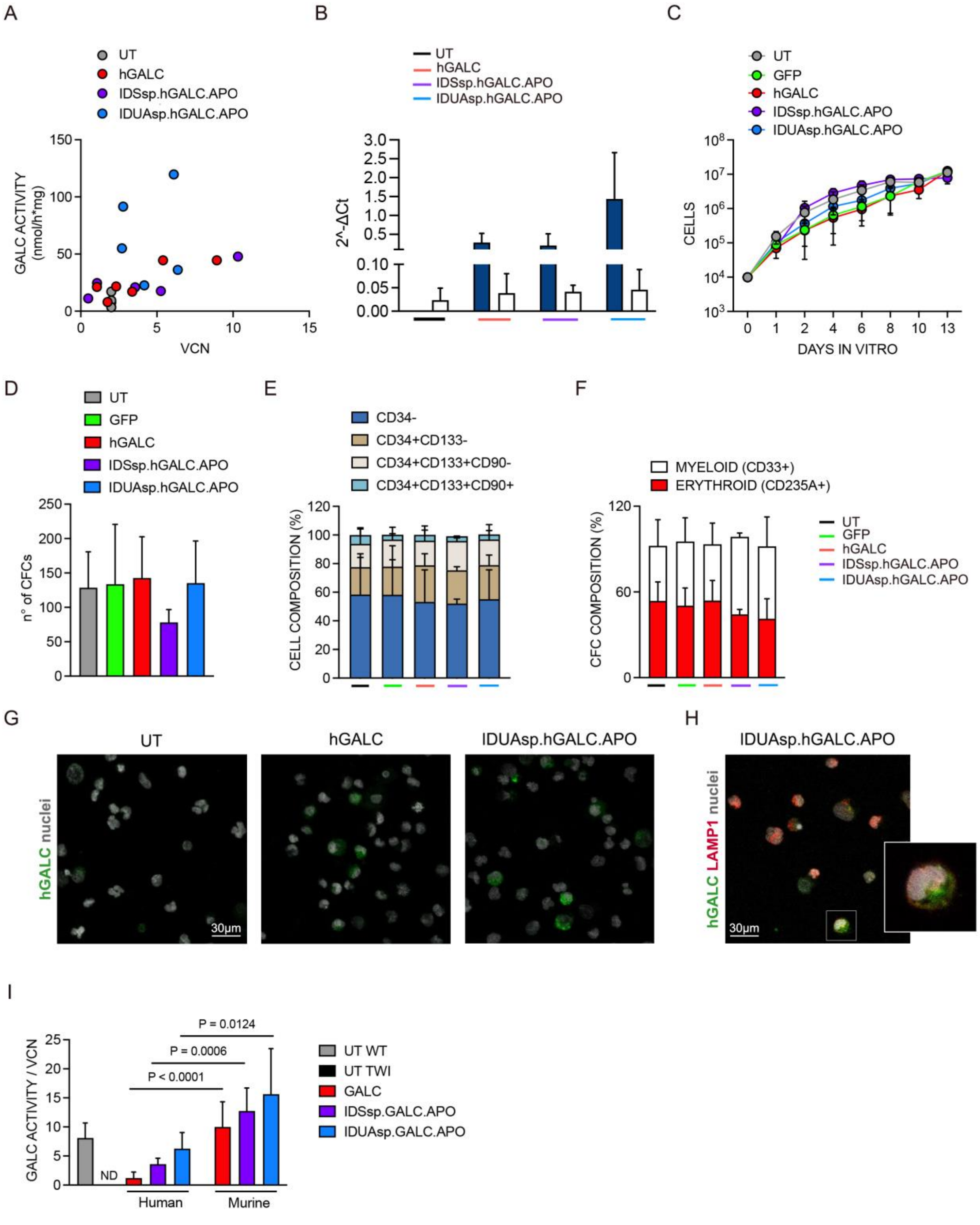

Figure S5. Safe and effective transduction of human CD34⁺ HSPCs with LVs expressing the native and chimeric GALC. **(A)** Correlation between GALC activity and Vector copy number (VCN) measured in LV-transduced CD34⁺ HSPC progeny (LC). n=5-6 experiments, 1-2 technical replicates/experiment. Spearman correlation, r = 0.7, p = 0.0033. **(B)** Transgenic (EX) and endogenous (END) GALC mRNA expression in UT and LV-transduced CD34⁺ HSPC progeny (LC). Data are expressed as mean (SD), $2^{-\Delta Ct}$ vs GAPDH (housekeeping gene), n=4-5 experiments, 2 technical replicates/experiment. **(C)** Growth curve of UT and LV-transduced CD34⁺ HSPCs. Data are expressed as the mean (SD), n=3-4 experiments, one replicate/experiment. **(D)** Number of colonies (CFC assay) originated from UT and LV-transduced CD34⁺ HSPCs. Data are expressed as the mean

(SD), n=5-6 experiments, 2 technical replicates/experiment. **(E)** Cell composition (expressed as percentage of the total number of cells analysed) of UT and LV-transduced $CD34^+$ HSPC populations assessed after 5 days of culture: mature blood cells (CD34-), early precursors ($CD34^+CD133^+CD90^+$), early progenitor cells ($CD34^+CD133^+CD90^-$), and lineage-committed progenitors ($CD34^+CD133^-$ $CD90^-$). Data are expressed as mean (SD), n=2-6 experiments, one technical replicate/experiment. **(F)** Percentage of $CD33^+$ (myeloid) and $CD235A^+$ (erythroid) colonies measured by FACS analysis. Data are expressed as mean (SD), n=2-6 experiments, one technical replicate/experiment. **(G)** Representative confocal images showing GALC protein expression (GALC; green) in UT and LV-transduced $CD34^+$ HSPC progeny (LC). Nuclei counterstained with Hoechst (grey). n=2 experiments, 2 coverslips/experiments. 40X magnification. Scale bar: 30 µm. **(H)** Representative image showing lysosomal localization (LAMP1, red) of the IDUAsp.hGALC.APO enzyme (GALC, green) in $CD34^+$ HSPC progeny (LC); Scale bar: 30 µm. Inset: magnification of one cell co-expressing the two markers. **(I)** GALC activity was measured in the pellet of TWI HSPC progeny (LC) transduced with LV.hGALC, LV.IDSsp.hGALC.APO, and LV.IDUAsp.hGALC.APO, as well as their murine counterparts. The data are normalized on the VCN and are presented as the mean (SD). Murine samples: n=6-9 experiments, human samples: n=3 experiments with 2 technical replicates/experiment. Statistical analysis was conducted using Welch's t-test

Table S1. Signal peptide sequence analysis. Percentage of basicity of the N-region and hydrophobicity of the H-region of selected lysosomal enzymes sp sequences retrieved using the Peptide 2.0 software (https://peptide2.com/N_peptide_hydrophobicity_hydrophilicity.php).

Lysosomal enzymes	Basicity of N-region (%)	Hydrophobicity of H-core (%)
Beta-Galactosylceramidase (GALC)	0	73.68
Iduronidase (IDUA)	42.86	93.75
Iduronate-2-sulfatase (IDS)	25	64.29
Arylsulfatase A (ARSA)	20	80
Hyaluronidase-1(HYAL-1)	25	78.57
Hexosaminidase A (HEXA)	20	78.57
Hexosaminidase B (HEXB)	0	72.73
Alpha glucosidase (GAA)	40	62.5
Alpha-galactosidase A (GLA)	22.22	68.42
Beta-galactosidase (GLB1)	14.29	75
Glucosamine (N-acetyl)-6-sulfatase (GNS)	20	57.69
Arylsulfatase B (ARSB)	20	77.78
Heparanase (HPSE)	0	64.25
Beta glucuronidase (GUSB)	20	81.25
Arylsulfatase G (ARSG)	0	80
N-acetyl-alpha-glucosaminidase (NAGLU)	0	73.33
Acid phosphatase 2, lysosomal (ACP2)	40	57.14
Mannosidase alpha class 2B member 1 (MAN2B1)	33.33	88.24
N-sulfoglucosamine sulfohydrolase (SGSH)	0	75
Heparan-alpha-glucosaminide N-acetyltransferase (HGSNAT)	16.67	65.38

Table S2. Titer and infectivity of LV batches.

Vector	Batch	Titer (TU/ml)	Infectivity (TU/ng)
LV.IDUAsp.mGALC.APO	#1	4.93E+09	1.80E+04
	#2	2.07E+09	4,32E+03
	#3	7.23E+09	3.92E+04
LV.mGALC	#1	1.22E+10	4.57E+04
LV.IDSsp.mGALC.APO	#1	1.32E+10	5.40E+05
	#2	4.57E+09	1.57E+04
LV.hGALC	#1	2.03E+09	4.84E+03
	#2	3.28E+09	9.97E+03
LV.IDSsp.hGALC.APO	#1	5E+09	1.2E+05
	#2	6.91E+09	2.4E+04
LV.IDUAsp.hGALC.APO	#1	5.25E+09	6.09E+04
	#2	1.34E+10	1.25E+04
	#3	3.87E+09	1.07E+04
LV.GFP	#1	2.93E+09	2.67E+04

Table S3. Primary and secondary antibodies.

Primary antibodies	Provider	Dilution			
		IF	*ImageStream*	*WB*	*FACS*
Polyclonal rabbit anti-calnexin	Sigma (c4731)	-	-	1:3,000	-
Hybridoma rat anti-Lamp1	DSHB (1D4B-S)	1:300	1:300	-	-
Monoclonal mouse anti-GalCer	Millipore (MAB342)	1:300	-	-	-
Monoclonal mouse anti-GFAP	Millipore (MAB3402)		-	1:100,0 00	-
Polyclonal rabbit anti-GFP	Thermo Fisher Scientific (A-11122)	1:1,000	-	-	-
Polyclonal rabbit anti-Iba1	Wako (019-19741)		-	1:1,000	-
Polyclonal rabbit anti-mCherry	Abcam (ab167453)	1:1,000	1:1,000	-	-
Monoclonal rabbit anti-mCherry	Abcam (ab213511)	-	-	1:1,000	-
Monoclonal human CD45 anti-mouse VioBlue	Miltenyi Biotec (130-110-802)	-	-	-	1:50
Monoclonal mouse anti-human CD34	BD Biosciences (348811)	-	-	-	1:10
Polyclonal rabbit anti-mouse GALC	Abcam (ab240638)			1:1,000	
Polyclonal rabbit anti-human GALC	Abcam (ab137750)	-	-	1:3,000	-
Anti-actin HRP	Sigma (a3854)	-	-	1:50,00 0	-
mouse anti-human GALC	kindly provided by Dr. J.E. Deane	1:30	-	-	-
Rabbit anti-Lamp1	Abcam (ab24170)	-	1:500	-	-
Mouse CD11b APC	Miltenyi Biotec	-	-	-	1:50
Anti-human CD133/2 PE	Miltenyi Biotec (130-113-186)	-	-	-	1:10
Anti-human CD90 APC	BD Biosciences (559869)	-	-	-	1:10
Anti-human CD19 PE	BD Biosciences (345789)	-	-	-	1:10
Anti-human CD45 APC-eFluor 780	eBioscience (47-0459-42)	-	-	-	1:10
Anti human CD38 perCP/Cy5.5	BioLegend (356614)	-	-	-	1:10
Anti-human CD33 BV421	BD Biosciences (562854)	-	-	-	1:10
Anti-human CD235 (Glycophorin A)	BD Biosciences (551336)	-	-	-	1:10
**Secondary antibodies**	**Provider**	**Dilution**			
		IF	*ImageStream*	*WB*	*FACS*
ALEXA 488 anti-mouse	Thermo Fisher Scientific (a11001)	1:1,000	1:1,000	-	-
ALEXA 488 anti-rabbit	Thermo Fisher Scientific (a11008)	1:1,000	-	-	-
ALEXA 546 anti-rabbit	Thermo Fisher Scientific (a11010)	1:2,000	-	-	-
ALEXA 594 anti-rabbit	Thermo Fisher Scientific (a11012)	1:2,000	-	-	-
ALEXA 647 anti-rat	Thermo Fisher Scientific (a21247)	1:500	1:500	-	-
HRP anti-mouse	Chemicon (AP124P)	-	-	1:5,000	-
HRP anti-rabbit	Chemicon (AP132P)	-	-	1:5,000	-
